# Clinical and microbiological features of host-bacterial interplay in chronic venous ulcers *versus* other types of chronic skin ulcers

**DOI:** 10.3389/fmicb.2023.1326904

**Published:** 2024-02-05

**Authors:** Mara Mădălina Mihai, Mircea Ioan Popa, Alina Maria Holban, Irina Gheorghe-Barbu, Liliana Gabriela Popa, Mariana-Carmen Chifiriuc, Călin Giurcăneanu, Coralia Bleotu, Corina Ioana Cucu, Veronica Lazăr

**Affiliations:** ^1^Department of Oncologic Dermatology–“Elias” University Emergency Hospital, “Carol Davila” University of Medicine and Pharmacy, Bucharest, Romania; ^2^Department of Botany-Microbiology, Faculty of Biology, Research Institute of the University of Bucharest, University of Bucharest, Bucharest, Romania; ^3^Department of Microbiology—“Cantacuzino” Institute, “Carol Davila” University of Medicine and Pharmacy, Bucharest, Romania; ^4^Cellular and Molecular Department, “Ştefan S. Nicolau” Institute of Virology, Bucharest, Romania

**Keywords:** chronic wound, host-microbiome, bacterial virulence factors, methicillin resistant *Staphylococcus aureus* (MRSA), tolerance at antimicrobials of biofilm cells, bio-marker

## Abstract

**Introduction:**

Chronic venous ulcers of the lower limbs develop in the context of advanced venous disease and have a significant impact on the patient’s quality of life, being associated with depression and worrisome suicide rates, as well as with an economic burden caused by increased medical care costs and high epidemiological risks of healthcare associated infections and emergence of strains resistant to multiple classes of antibiotics and/ or antiseptics. Although numerous studies have investigated the composition of the chronic wounds microbiome, either by culture-dependent or independent methods, there are no data on the association between virulence and resistance profiles of strains isolated from venous ulcers and the clinical picture of this pathology. The elucidation of pathogenic mechanisms, at both phenotypic and molecular level, is crucial in the fight against these important human microbial agents, in order to develop novel biomarkers and discover new therapeutic targets.

**Methods:**

In this study we aimed to characterize the phenotypic virulence profiles (including the ability to develop biofilms) of microorganisms isolated from chronic skin wounds and to correlate them with the clinical symptomatology. Considering the high incidence of *Staphylococcus aureus* infections in chronic ulcers, but also the ability of this species to develop multi-drug resistance, we performed an more in-depth study of the phenotypic and genotypic virulence profiles of methicillin-resistant *Staphylococcus*.

**Results:**

The study revealed important differences regarding the clinical evolution and virulence profiles of microorganisms isolated from lower limb wounds, as well as between patients diagnosed with chronic venous ulcers and those with lesions of different etiology.

## 1 Introduction

Most chronic wounds of the lower limbs are of venous etiology (70%–90%; Kahle et al., 2011; Stanek et al., 2023). Chronic venous ulcers of the lower limbs are defined as wounds that develop in the context of advanced venous disease, with a slow healing rate and no tendency to heal over 3 months despite adequate treatment or not fully healed at 12 months (Kahle et al., 2011; Stanek et al., 2023). They have a significant impact on the patient’s quality of life, being associated with depression and worrisome suicide rates, as well as an economic burden due to increased medical care costs (dressings, hospitalization, and others; Kahle et al., 2011; Weller et al., 2021; Stanek et al., 2023). Moreover, they imply an epidemiologic risk. In the context of recurrent hospitalizations and long-term antibiotic treatment, a worrisome issue arises both in Romania, as well as at global level: healthcare associated infections and the emergence of strains resistant to multiple classes of antibiotics and/ or antiseptics (Rhoads et al., 2012; Salavastru et al., 2012; Mihai et al., 2014), with medical and socio-economic disastrous consequences. Therapeutic approaches are limited, having an important impact on the chances of healing and/or survival.

Although the exact pathogenicity of delayed healing in chronic venous ulcers is unknown, several factors have been described: endogenous (e.g., vascular disease, comorbidities, immunity of the host, the presence of vicious scars after local trauma or surgery) or exogenous (e.g., bacterial colonization or infection, local therapeutic agents that impair healing, excessive hygiene; Cooper et al., 2014; Percival et al., 2014; Weller et al., 2021). Marques et al. (2023) reviewed the prognostic factors associated with delayed healing of wounds in adults: male gender, sedentarism with the decline in activities of daily life, history of ulcers, comorbidities such as renal disease, diabetes, and peripheral arterial disease, wound characteristics such as duration, area affected, and location, and wound complications such as high-stage WIfI (Wound, Ischemia, foot Infection) classification, gangrene, infection, and low ankle brachial index.

According to the International Wound Infection Institute Wound Infection Continuum (IWII-WIC) there are different, progressively more severe phases of microbial presence in a wound, ranging from contamination to critical colonization, local infection, spreading infection (cellulitis) defined by inflammation or erythema greater than two centimeters from the wound edge, and systemic infection/sepsis (International Wound Infection Institute (IWII), 2022). A local infection is a stage when wound bacteria proliferate and activate host immune defense mechanisms, leading to local tissue damage and impaired healing [International Wound Infection Institute (IWII), 2022]. Local infection causes changes within the wound (hypergranulation and/or friable granulation, bleeding, epithelial bridging and pocketing, increased exudate, purulent discharge, delayed wound healing beyond expectations, new or increasing pain, and/or increasing malodor) and its immediate skin surroundings [erythema, local warmth, swelling, pain; International Wound Infection Institute (IWII), 2022].

Every open wound has bacteria colonizing it, however not every contaminated wound gets infected. The main factors that influence the development of wound infection are represented by: i) the immune status of the host and, implicitly, the capacity to combat opportunistic pathogens; ii) the wounds’ bacterial load, since more microorganisms have a better chance to counteract the host’s immune defense mechanisms; iii) the contaminant bacterial strains, since some are more virulent, including a higher ability to develop biofilms; iv) the polymicrobial associations, since some microorganisms appear to develop synergistic effects [social microbiology; International Wound Infection Institute (IWII), 2022]. Patients suffering from systemic or local immunosuppression (e.g., advanced *Human immunodeficiency virus* infection, the use of medication in organ recipient patients, the topical preoperative corticosteroid treatment, others) are more susceptible to delayed wound-healing and wound-related complications issues (Alqatawni et al., 2020; Varga et al., 2021).

Chronic wound bacteria impede normal healing by several mechanisms: microbial invasion, the development of virulence traits that increase the pathogenicity of bacteria (e.g., production of soluble virulence factors, the development of mono- or polymicrobial biofilms), resistance and/ or tolerance to antimicrobial agents, and to host immune defense mechanisms. Moreover, bacterial synergism in polymicrobial wound infections (e.g., *Staphylococccus aureus*, *S. aureus* and *Pseudomonas aeruginosa*, *P. aeruginosa*) promotes the virulence and persistence of infection, as well as a decreased response to therapy (DeLeon et al., 2014).

The virulence of bacteria is a context dependent, multifactorial, and dynamic property (Diard and Hardt, 2017). There are very few studies that have correlated the virulence profile of bacteria isolated from different types of chronic infections with the host immune response or with the clinical outcome. Sotto et al. (2008) studied the virulence potential of 132 *S. aureus* strains isolated from diabetic foot ulcers. They showed that a particular combination of virulence genes (*cap8*, *sea*, *sei*, *lukE*, and *hlgv*) was statistically correlated with infection and may serve as a prediction factor for the wound status at the follow-up (Sotto et al., 2008). Moreover, Wu et al. (2023) demonstrated that the levels of glucose and its metabolites impact on the virulence and inflammatory response of *S. aureus* strains isolated from diabetic foot ulcers. Regarding chronic venous ulcers, Cwajda-Białasik et al., (2021) proved that culture-positive lesions, and, therefore, colonization or infection status, are more frequent in older patients (age > 65 years) with chronic and larger wounds (ulcer duration > 12 months, ulceration area > 8.25 cm^2^). Different from the studies of diabetic foot ulcers, Gajda et al. (2021) did not observe a significant correlation between the virulence of *S. aureus* strains isolated from venous ulcers and the presence of certain genes, in particular, between *pvl* genes and the *spa* type.

*Staphylococcus aureus* is the most frequent species isolated from chronic wounds and it is well known that methicillin-resistant *S. aureus* (MRSA) causes severe infections. The strains persist in the hospital environment, where, under the selective pressure of antibiotics, they evolve, with the expression of β-lactamase coding genes, as well as some metabolic and virulence genes. Moreover, they can transfer resistance genes to other species directly or indirectly. Besides antibiotic resistance, *S. aureus* triggers disease through various pathogenic mechanisms, involving both exotoxins, including pore-forming toxins, staphylococcal superantigens, as well as bacterial adhesins and biofilm development (Baba et al., 2002).

Although numerous studies have investigated the composition of the microflora of chronic wounds, either by classical diagnostic methods or by combining molecular methods, there are few comparative data on the virulence and resistance profiles of strains isolated from venous ulcers with the manifested clinical picture. The elucidation of pathogenic mechanisms, at the molecular level, is crucial in the fight against these important human microbial agents, in order to discover new therapeutic targets.

In this study we aimed to characterize the phenotypic virulence profiles (including the ability to develop biofilms) of microorganisms isolated from chronic skin wounds (venous, arterial and mixed arterio-venous ulcers, pressure sores, wounds secondary to surgery or associated with abscesses, paraneoplastic ulcerations or secondary to autoimmune vesiculo-bullous diseases—pemphigus vulgaris, bullous pemphigoid) and to correlate the data obtained with the clinical picture. Considering the high incidence of *S. aureus* infections at the level of chronic ulcers, but also the problem of developing multi-drug resistance, we also performed an in-depth study of this bacterial species, by analyzing the phenotypic and genotypic virulence profile of MRSA strains.

## 2 Materials and methods

We performed an observational, prospective study that included 80 patients diagnosed with chronic skin wounds (39 venous ulcers, 41 other types of chronic skin wounds), showing clinical signs of infection, hospitalized in the “Elias” Emergency University Hospital, Bucharest, Romania, during the last 5 years. Other types of chronic skin wounds were: arterial ulcers (Cooper et al., 2014), mixed arterio-venous ulcers (Rhoads et al., 2012), pressure sores (Mihai et al., 2014), wounds secondary to surgery (Weller et al., 2021) or associated with abscesses (International Wound Infection Institute (IWII), 2022), paraneoplastic ulcerations (Kahle et al., 2011) or secondary to autoimmune vesiculo-bullous diseases—pemphigus vulgaris, bullous pemphigoid (Marques et al., 2023). There were excluded patients who received systemic antibiotic treatment during the past month or topical antimicrobials during the past week before hospital admission, who had a diagnosis of immunosuppression or under treatment with immunosuppressive drugs/medication. Also, there were excluded the cases where there were lacking clinical criteria of wound infection (Bianchi et al., 2016; International Wound Infection Institute (IWII), 2022).

Monitoring of patients began on the date of the diagnosis of a chronic, superinfected wound. The follow-up was performed for all patients at 1 month and at 6 months after diagnosis, analyzing the clinical evolution, including the status of a healed wound (complete epithelialization). The follow-up was objectified by the RESVECH 1.0 scale and by non-invasive imaging through serial comparative photography. Patients with more frequent hospitalizations were evaluated each time. The follow-up period ended 6 months after diagnosis, in the case of skin healing, or 1 year in patients with persistent skin lesions.

An informed consent was obtained from each patient. The study protocol is in accordance to the ethical prerogatives of the 1975 Declaration of Helsinki, respecting the Good Clinical Practice (GCP) hospital admission, standards to the same extent, and was approved by the Ethics Commission of the “Elias” University Emergency Hospital, Bucharest.

The laboratory tests were performed at the “Elias” Emergency University Hospital, the Institute of Research of the University of Bucharest, Romania and the “Cantacuzino” National Medico-Military Institute for Research and Development. The identity of the bacterial strains was confirmed using classical microbiological phenotypic methods, automated Vitek 2 system and Phoenix BD system. Several phenotypic and genotypic virulence and antibiotic resistance features of the bacterial strains were evaluated.

### 2.1 Characteristics of the host

Clinical data was collected by anamnesis (the etiology, location and the presence of clinical signs of infection for each chronic wound, the patient’s history of associated aggravating pathologies), full body examination, and wound assessment. In order to establish a positive diagnosis, studies of arterial and venous supply were performed in patients with chronic wounds of the lower limbs (ultrasound examination, ankle-brachial index). Chronic venous disease was classified according to the CEAP classification and the Venous Clinical Severity Score (Lurie et al., 2020).

If there was a clinical suspicion of neoplasm, inflammatory or autoimmune disease (e.g., vesiculo-bullous disorder) or if the skin ulcers were refractory to 3 months or more of appropriate treatment, a wound biopsy sample was taken, followed by histopathologic examination and/ or direct immunofluorescence assessment (Senet et al., 2012; Isoherranen et al., 2019; Erfurt-Berge et al., 2023).

Wound severity and progression were objectively assessed by serial imaging and by the application of RESVECH 1.0 clinical scale (Resultados Esperados de la Valoración y Evolución de la Cicatrización de las Heridas Crónicas 1.0; minimum value 0, maximum value 40, Restrepo-Medrano, 2011; Cruz et al., 2023). Therefore, each wound was evaluated according seven main items: surface, depth and affected tissues, condition of the edges of the lesion, type of tissue in the wound bed, type of exudate, signs of infection/inflammation (increasing pain, perilesional erythema, perilesional edema, high temperature, increasing exudate, purulent exudate, friable tissue or tissue that bleeds easily, stagnant wound non-progressive tissue, biofilm-compatible tissue, odor, hyper-granulation, increased wound size, satellite lesions, tissue pallor), and incidence of pain (Restrepo-Medrano, 2011; Cruz et al., 2023).

Laboratory markers of inflammation and/or infection were also assessed (complete blood count, erythrocyte sedimentation rate, C-reactive protein, fibrinogen).

### 2.2 Microbiological characterization

#### 2.2.1 Strain identification

After wound cleaning with physiological serum and debridement, samples for bacterial culture were obtained by rotating a sterile swab over a 1-cm^2^ region of the wound, with pressure to express exudate, following the Levine approach. This method was also recommended by the IWII-WIC consensus—“Wound Infection in Clinical Practice” [International Wound Infection Institute (IWII), 2022], since it allows the sampling of a higher concentration of bacteria both from the wound’s surface, as well as from its depth (Angel et al., 2011).

Wound swabs were tested using routine aerobic culture techniques. Specimens were Gram stained. Pure colonies were obtained by inoculating each swab onto 5% sheep blood agar, Chocolate agar, MacConkey agar (without crystal violet) and Sabouraud agar with chloramphenicol (Oxoid). All plates were incubated aerobically at 37°C for 18–24 h, with the exception of Sabouraud plates-incubated simultaneously at 30°C and 37°C for 24–72 h. The biochemical identification was performed using the automated Vitek 2 system (bioMérieux) and Phoenix BD (Bekton–Dickinson). Commensal bacteria (coagulase-negative staphylococci) were excluded from the study. The susceptibility to antibiotics was assessed using disk diffusion method according to the CLSI guideline (*Clinical and Laboratory Standards Institute*) and automated systems (Vitek2C/Phoenix BD). D-tests were also performed to evaluate the inducible resistance to clindamycin, following the manufacturer’s recommendations (Magiorakos et al., 2012). Subsequently strains were maintained at 4°C in the Microbial Culture Collection of Microbiology Laboratory, Faculty of Biology, Bucharest. For further experiments, strains were streaked on nutrient agar and incubated over night at 37°C.

#### 2.2.2 Phenotypic assessment of the virulence profile of microorganisms

##### 2.2.2.1 Phenotypic assessment of the adherence to human cell substrate

Bacterial adherence to HeLa and endothelial cells was performed by the adapted Cravioto’s method (Cravioto et al., 1979; Lazăr, 2003; Bleotu et al., 2010; Holban et al., 2013; Curutiu et al., 2014).

**Figure 1:**
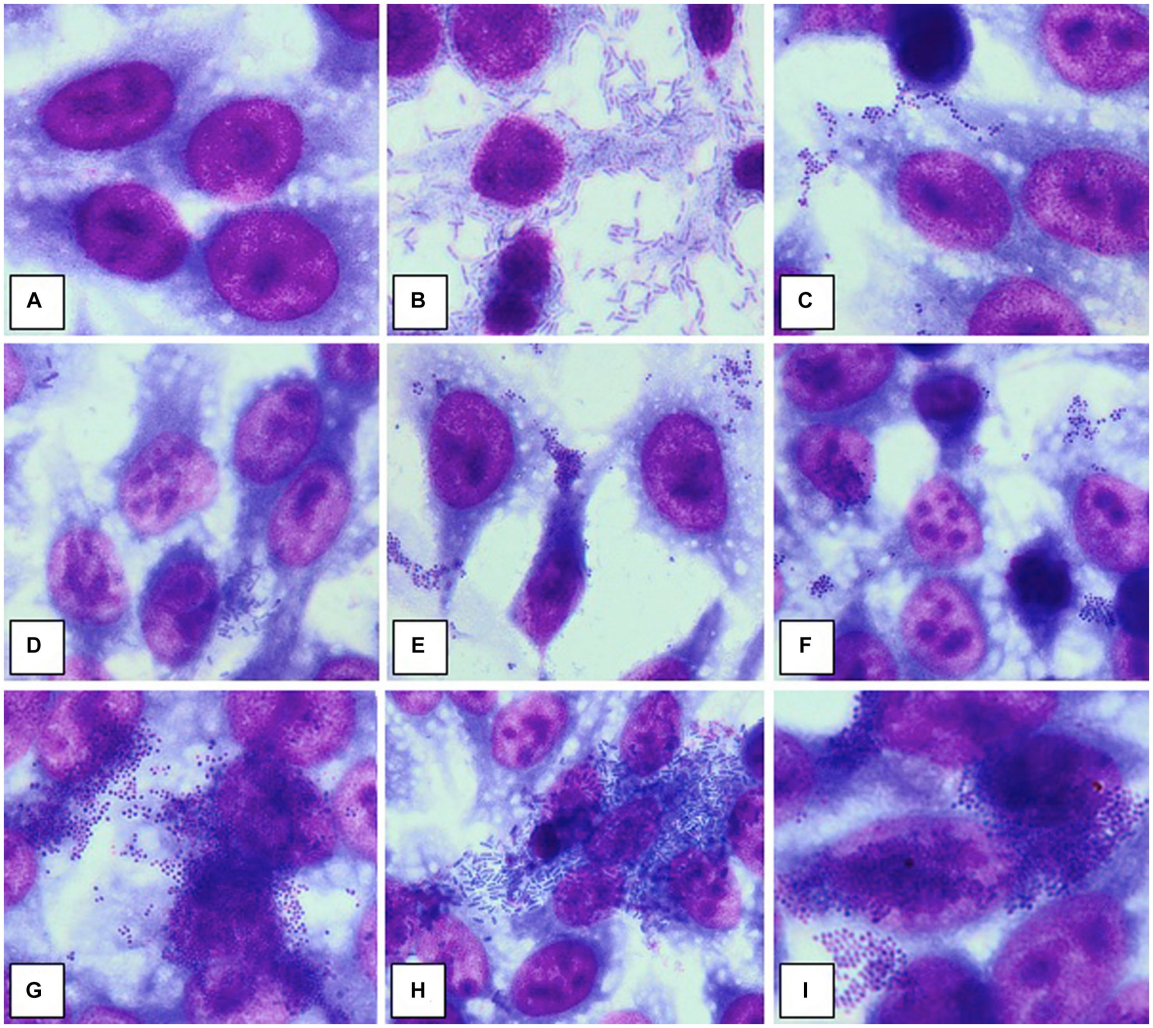
Adherence patterns to HeLa cell substrate. **(A)** No adherence; **(B)** Diffuse aggregative adherence in a strain of *Pseudomonas aeruginosa*; **(C)** Diffuse adherence in a strain of *Staphylococcus aureus*; **(D)** Aggregative localized adherence, in a strain of *Escherichia coli*; **(E)** Aggregative localized adherence, in a strain of *Staphylococcus aureus*; **(F)** Aggregative localized adherence, in a strain of methicillin-resistant *Staphylococcus aureus*; **(G)** Aggregative diffuse adherence, in a strain of methicillin-resistant *Staphylococcus aureus*; **(H)** Aggregative diffuse adherence, in a strain of *Klebsiella pneumoniae*; **(I)** Aggregative diffuse adherence, in a strain of methicillin-resistant *Staphylococcus aureus*. Source: Mihai et al. (2014). Published with the permission of authors.

Briefly, the HeLa or endothelial diploid cell monolayers were washed three times with phosphate buffered saline (PBS) and then, 1 mL of fresh medium without antibiotics was added to each well. Each well was then seeded with 1 ml of bacterial suspension of each strain prepared from bacterial mid-logarithmic phase cultures grown in nutrient broth and adjusted to 10^7^ CFU/mL. The inoculated plates were incubated for 2 h at 37°C. The adherence patterns were defined as: localized adherence (LA), when individual microbial cell clusters were observed on the surface of HeLa/endothelial cells; localized aggregative adherence (LAA), when the localized aggregates showed a layered adherence pattern, like “bricks in a wall”; diffuse adherence (DA), when the bacteria adhered diffusely, covering the entire HeLa/ endothelial cell surface; diffuse aggregative adherence (DAA), when the bacteria adhered diffusely, covering the entire cellular surface, having a layered adhesion pattern, like “bricks in a wall” (Figure 1; Cravioto et al., 1979; Lazăr, 2003; Bleotu et al., 2010; Holban et al., 2013; Curutiu et al., 2014; Mihai et al., 2014). For each strain, it was established an adherence index defined by the ratio between the number of HeLa/endothelial cells to which microorganisms adhered and 100 eukaryotic cells counted on the microscopic field (Cravioto et al., 1979; Lazăr, 2003; Bleotu et al., 2010; Holban et al., 2013; Curutiu et al., 2014; Mihai et al., 2014).

##### 2.2.2.2 Phenotypic assessment of biofilm development *in vitro*

The ability of the isolated strains to produce biofilm was evaluated using the violet crystal microtiter method, with the spectrophotometric quantification of the biomass adhered to the inert substrate of 96-well plates. Overnight bacterial cultures were diluted in Tryptic Soy Broth, up to a turbidity of 0.5 McFarland (approximately 1 × 10^8^ CFU/mL) and 20 μL of the obtained suspension were seeded in 96 multi-well plates in a volume of 180 μL liquid medium in triplicate. To allow biofilm formation, the inoculated plates were incubated for 24, 48, and 72 h at 37°C. After each incubation period, the biofilms were gently washed with PBS with the aim of removing planktonic cells. The adhered biofilm mass was treated with cold methanol for 5 min, dried at room temperature dried at room temperature, stained with stained with 0.1% crystal violet solution for 15 min, resuspended in 33% acetic acid, and the absorbance spectrophotometrically read at 492 nm. The quantity of adhered biomass is proportional to the absorbance value (Coffey and Anderson, 2014; Mihai et al., 2014; Preda et al., 2021).

##### 2.2.2.3 Phenotypic assessment of the production of soluble virulence factors

**Figure 2:**
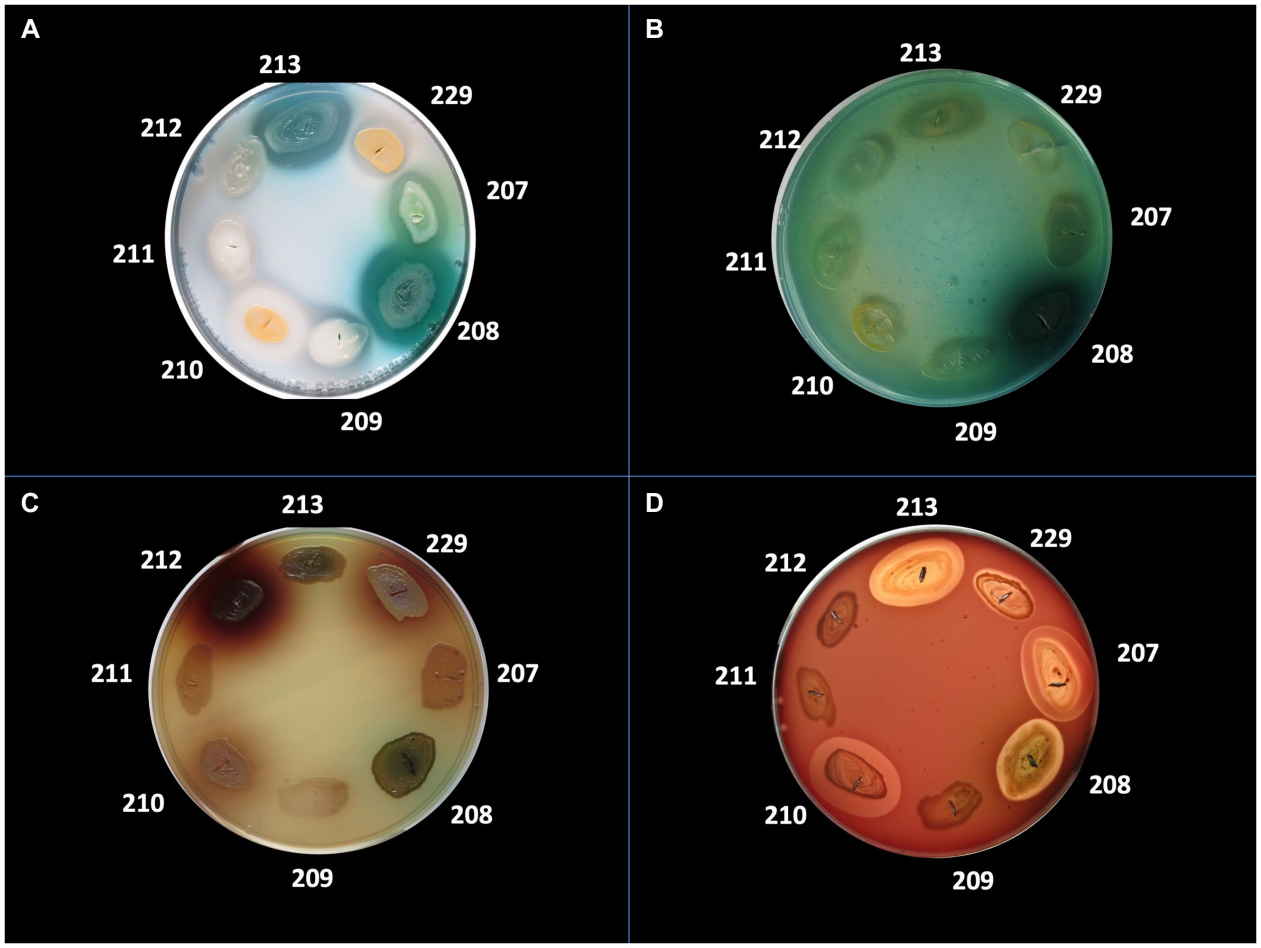
Detection of the production of different virulence factors in 8 bacterial strains analyzed. **(A)** Virulence factor: caseinase, medium: agar with addition of 15% casein; **(B)** Virulence factor: DNase, medium: DNase; **(C)** Virulence factor: esculinase, medium: agar supplemented with 1% esculin and iron citrate; **(D)** Virulence factor: hemolysins, medium: agar supplemented with 5% sheep blood. Bacterial strains tested: no. 207 *Pseudomonas aeruginosa*, no. 208 *P. aeruginosa*, no. 209 *Escherichia coli*, no. 210 *Staphylococcus aureus*, no. 211 MRSA, no. 212 *Klebsiella pneumoniae*, no. 213 *P. aeruginosa.*

**Figure 3:**
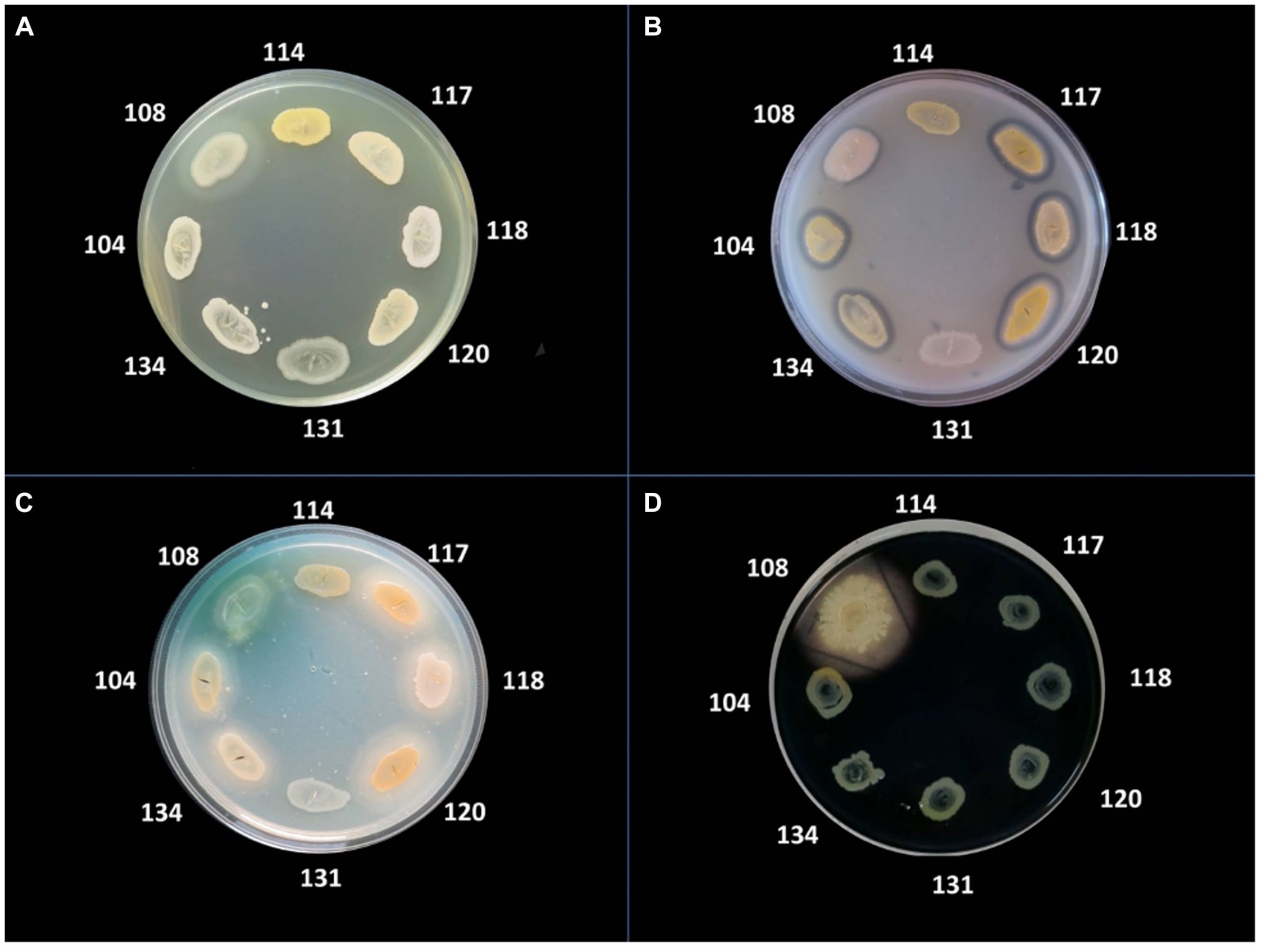
Detection of the production of different virulence factors in 8 bacterial strains analyzed. **(A)** Virulence factor: gelatinase; medium: agar with the addition of 3% gelatin; **(B)** Virulence factor: lecithinase, medium: agar with the addition of 2.5% egg yolk; **(C)** Virulence factor: lipase, medium: agar supplemented with 1% Tween 80; **(D)** Virulence factor: amylase, medium: agar with 1% starch added, flooded with Lugol solution. Bacterial strains tested: no. 104 *Staphylococcus aureus*, no. 108 *Pseudomonas aeruginosa*, no. 114 MRSA, no. 117 *S. aureus*, no. 118 *S. aureus*, no. 120 *S. aureus*, no. 131 *S. aureus*, no. 134 MRSA.

The bacterial virulence phenotype was assessed by performing enzymatic tests for the expression of eight soluble factors, using the following specific media: 5% sheep blood agar (for alpha and beta hemolysins), 2.5% yolk agar (lecithinase test), 1% Tween 80 agar (lipase test), 15% casein agar (caseinase test), 1% gelatin agar (gelatinase), 10% starch agar (amylase), DNA agar (DN-ase test), 1% esculin iron salts (esculinase test) (Cravioto et al., 1979; Bleotu et al., 2010; Holban et al., 2013; Curutiu et al., 2014; Preda et al., 2021). Nutritive agar base was supplemented with various substrata and biochemical indicators to allow for the detection of particular bacterial enzymes. From the 24 h bacterial culture a bacterial inoculum of McFarland 0.5 (1.5 × 10^8^ CFU/mL) was prepared and was spot inoculated with a 10 μL sterile loop in Petri dishes with specific media (Cravioto et al., 1979; Bleotu et al., 2010; Holban et al., 2013; Curutiu et al., 2014; Preda et al., 2021). The strains were incubated for 24 h at 37°C and at 25°C for the next 48 h to allow the production and observation of specific enzymatic virulence factors, evaluated after 24, 48, and 72h of incubation (Cravioto et al., 1979; Bleotu et al., 2010; Holban et al., 2013; Curutiu et al., 2014; Preda et al., 2021). The results were objectified by observing the change of culture medium aspect following the enzymatic reaction: for alpha and beta hemolysins test, the haemolytic zone surrounding the inoculation spot; for the lecithinase, lipase, caseinase, and gelatinase production, the opaque/ precipitation zone surrounding the culture spot; for DN-ase reaction, the change of color from light green to pink in the area surrounding the spot culture; for the esculinase test, the black precipitation zone surrounding the culture spot; amylase production was noted after adding iodine solution (Cravioto et al., 1979; Bleotu et al., 2010; Holban et al., 2013; Curutiu et al., 2014; Preda et al., 2021). The virulence factors production was evaluated with a score from 0 to 4 (0 was the minimum, while 4 was the maximum), depending on the diameter of the culture medium area of change around the culture spot (as exemplified in Figures 2, 3; Cravioto et al., 1979; Bleotu et al., 2010; Holban et al., 2013; Curutiu et al., 2014; Preda et al., 2021).

#### 2.2.3 Genotypic assessment of virulence genes of MRSA strains by polymerase chain reaction

The genotypic virulence profiles were assessed by PCR for 24 MRSA strains. The molecular reactions were performed using the Corbet Thermal Cycler. Amplification products for each PCR reaction (simplex or multiplex) were visualized by electrophoresis in 1% agarose gel, stained with SYBR Safe DNS (Thermo Scientific, Bucharest, Romania) and identified based on characteristic sizes using specific molecular weight markers (M-Bench Top 100 bp DNA Ladder, Promega, United States; Preda et al., 2021).

Bacterial genomic DNA was extracted by the alkaline method. 1–5 MRSA colonies were suspended in 20 μL NaOH (0.05 M) and sodium dodecyl sulfate (0.25%) solution. In the next step, amplification was performed at 95°C for 15 min, followed by the addition of 180 μL TE1x buffer solution to each Eppendorf tube and centrifugation for 3 min at 13,000 r.p.m. The purity and concentration of the obtained DNA product was checked by electrophoresis in 1.5 and 2% agarose gel, 45 min at 90 V, stained with SYBR Safe DNS (ThermoScientific, Bucharest, Romania). The genomic DNA extract was stored at −4°C and used as template for all PCR experiments (Preda et al., 2021).

The genotypic virulence profile for 24 MRSA strains was characterized by analyzing the presence of genes encoding eight virulence factors, intensively expressed in these selected strains.

The protocol developed by Cotar et al. (2010) was followed, respecting the sequences of the primers used as well as the reaction parameters. Several virulence genes were detected by Polymerase Chain Reaction (PCR) through uniplex/simplex tests (*fnbA* gene coding, fibronectin adhesin A; *coag* gene, coagulase enzyme) and multiplex tests (*clf*A and *clf*B genes, bacterial surface adhesins; *fnb*B gene, fibronectin binding protein B; *fib* gene, fibrinogen binding protein; *bbp* gene, bone sialoprotein–binding protein; *ebpS*, elastin binding protein; Cotar et al., 2010). The reaction mix used was GoTaq® Green Master Mix (Jena Bioscience, Germany; Cotar et al., 2010).

### 2.3 Statistical analysis

The obtained results were analyzed statistically. The correlation between the continuous variables was tested by estimating the Pearson linear correlation coefficients (r) according to the established criteria. Correlation coefficients can take values between −1 and 1, showing a negative correlation (between −1 and 0) or a positive correlation (between 0 and 1). Statistical analysis was performed using Excel and Analyse-it (Method Validation Edition) software.

## 3 Results

### 3.1 Characteristics of the host

#### 3.1.1 General characteristics of the patients’ cohort

The study cohort was represented by 80 patients diagnosed with chronic skin wounds split in group no. 1 of 39 venous ulcers and group no. 2 of 41 other types of chronic skin wounds (7 arterial ulcers, 6 arterio-venous ulcers, 4 pressure sores, 3 post-surgical wounds, 10 wounds associated with abscesses, 2 paraneoplastic ulcerations, 9 ulcerations in the context of autoimmune vesiculo-bullous diseases) hospitalized in the “Elias” Emergency University Hospital, Bucharest, Romania, during the last 5 years.

From the total number of patients, 42 were females, 38 males, with a mean age of 62.1 and median age of 65.25 years old, while in the group suffering from chronic venous ulcers there were 23 female and 16 male patients, with a mean age of 69.1 and median age of 70 years old (Table 1).

**Table 1:** Mean, median, and standard deviation values of age in patients depending on diagnosis and gender.

All types of ulcers	Total (*n* = 80)			Female (*n* = 42)			Male (*n* = 38)		
	Mean	Median	SD	Mean	Median	SD	Mean	Median	SD
Age	62.1	65.25	18.6	61.23	64	19.16	62.97	66.5	18.17

Venous ulcers	Total (*n* = 39)			Female (*n* = 23)			Male (*n* = 16)		
	Mean	Median	SD	Mean	Median	SD	Mean	Median	SD
Age	69.1	70	12.55	69.04	70	12.36	69.18	68	13

Other types of chronic skin wounds	Total (*n* = 41)			Female (*n* = 19)			Male (*n* = 22)		
	Mean	Median	SD	Mean	Median	SD	Mean	Median	SD
Age	55.36	59	21.02	51.78	49	21.97	58.45	63.5	20.16

It was registered a total number of 147 hospitalizations of the enrolled patients, more frequent during summer compared to other seasons (67 vs. 80), with an average of two times per patient and with a maximum of 14 hospitalizations for chronic venous wounds.

Regarding the comorbidities, in the group suffering from chronic venous ulcers the most frequent ones were essential hypertension (72%), ischemic cardiomyopathy (56.4%), dyslipidemia (41%), and type I/II diabetes (38.5%). 46.2% had a history of deep vein thrombosis of the lower limbs. The prevalence of comorbidities in patients diagnosed with venous ulcers and with other types of chronic lower limb wounds (arterio-venous ulcer, arterial ulcer) were comparatively analyzed. Patients with arterio-venous ulcers presented in a significantly higher proportion cardiac arrhythmia (66.7% vs. 30.8%), cardiac valvopathy (66.7% vs. 35.9%), ischemic cardiomyopathy (66.7% vs. 56.4%), hepatic steatosis (16.7% vs. 7.7%) and less frequently diabetes mellitus (16.7% vs. 38.5%), dyslipidemia (33.3% vs. 41%). Compared to patients diagnosed with chronic venous ulcers, patients with arterial ulcers had a significantly higher frequency of hepatic steatosis (28.6% vs. 7.7%), type I/II diabetes (42.9% vs. 38.5%), dyslipidemia (42.9% vs. 41%) and less hypertension (28.6% vs. 72%), ischemic cardiomyopathy (42.9% vs. 56.4%), cardiac arrhythmia (14.3% vs. 30.8%), and cardiac valvulopathy (28.6% vs. 35.9%).

#### 3.1.2 Clinical characteristics of the wounds

##### 3.1.2.1 Wound size

Depending on their size, the wounds were classified according to the RESVECH questionnaire, in the following six intervals: 1 = 0.1-4 cm^2^, 2 = 4–15.9 cm^2^, 3 = 16–35.9 cm^2^, 4 = 36–63.9 cm^2^, 5 = 64–99.9 cm^2^, 6= > 100 cm^2^. 15 patients presented venous ulcerations larger than 16 cm^2^ and only one venous ulcer exceeded 100 cm^2^. The majority of the patients with chronic venous ulcers were included in the first three intervals, with the highest number in the second one of 4–15.9 cm^2^ (Table 2).

**Table 2:** Wound size classified in six intervals.

Wound size	0.1–4 cm^2^	4–15.9 cm^2^	16–35.9 cm^2^	36–63.9 cm^2^	64–99.9 cm^2^	>100 cm^2^	Total
Venous ulcer	7	17	9	2	3	1	39
Arterial ulcer	0	5	1	0	5	0	11
Arterio-venous ulceration	1	2	2	0	0	1	6

##### 3.1.2.2 RESVECH score

The RESVECH score (minimum value 0, maximum value 40) in patients with chronic venous ulcers (Group 1) ranged from 12 to 34, with a mean of 21.61 and a median of 19. The venous wounds were exceeded in severity by arterial ulcers with a median RESVECH of 21.5, by arterio-venous ulcers with 24, and pressure sores with 25, values correlated with the median surface area of each type of lesion and with the median evolution (Table 3). The Pearson linear correlation test of the size of the ulceration and the RESVECH score, in patients with venous ulcers, issued a correlation coefficient r = 0.837 > 0.28, highlighting an intensely positive correlation between the two variables.

**Table 3:** Mean, median, and standard deviation values of the RESVECH score in patients depending on diagnosis.

	RESVECH score		
	Mean	Median	SD
Venous ulcers (*n* = 39)	21.61	19	4.88
Arterial ulcers (*n* = 7)	22.14	21.5	3.38
Arterio-venous ulcers (*n* = 6)	24.6	24.5	6.28
Pressure sore (*n* = 4)	25	25	0

#### 3.1.3 Systemic inflammatory response

Although the inflammatory biological syndrome was present in 59% of the patients diagnosed with chronic venous ulcers (Group 1), only 20.5% associated leukocytosis with neutrophilia. Compared to venous ulcers, in the case of other types of chronic skin lesions (Group 2) a lower proportion of biological inflammatory syndrome (51.2%) was observed, to which it was associated more frequently (approximately twice) leukocytosis with neutrophilia (41.5%). This may reflect the fact that the host response to infection may be more severe in other types of chronic skin lesions compared to venous ulcers. The Pearson test of the linear correlation demonstrated a weak statistically positive correlation between the presence of the biological inflammatory syndrome detected at the initial monitoring and the evolution of skin wounds, with a Pearson r value of 0.234 < 0.28.

#### 3.1.4 Evolution of disease

**Figure 4:**
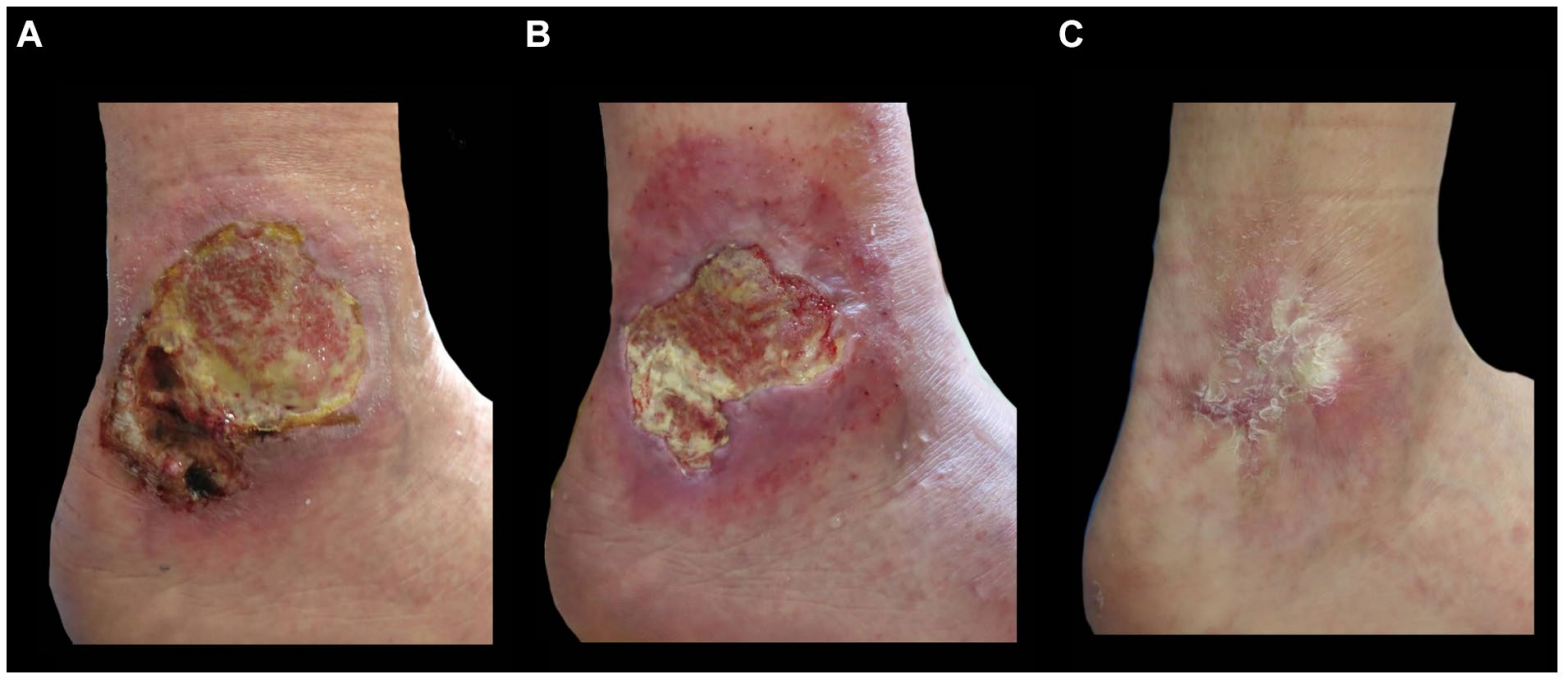
Favorable evolution of a patient diagnosed with chronic venous leg ulcer. **(A)** Initial clinical aspect of the wound. **(B)** Clinical aspect at 2 months. **(C)** Clinical aspect at 6 months.

One third of the total number of patients diagnosed with chronic venous ulcers (Group 1; 33.33%) was cured (Figure 4), this proportion being significantly higher compared to that observed in leg ulcers of other etiology, as well as in the case of arterial wounds (14.28%), mixed etiology (0%). However, 71.4% of patients with arterial ulcers had a favorable evolution, with a reduction in the RESVECH score. Ulcers of mixed etiology (arterio-venous) presented a more severe evolution, with stationary or aggravated lesions at the end of the monitoring period.

A higher proportion of severe septicemic events resulting in death were observed in patients diagnosed with other types of chronic skin lesions (Group 2) compared to patients with venous ulcers (Group 1; Weller et al., 2021; Stanek et al., 2023).

In patients with venous leg ulcers (Group 1), the Pearson test of linear correlation did not demonstrate a statistically relevant correlation between the evolution of the ulceration at the end of monitoring and its initial size (Pearson r = 0.052), respectively the initial RESVECH score (Pearson r = 0.037).

### 3.2 Microbiological characterization

#### 3.2.1 Strain identification

A total number of 104 bacterial strains were isolated from 80 patients diagnosed with chronic skin lesions, admitted to the “Elias” Emergency University Hospital, Bucharest, Romania, during the last 5 years. 49 strains were isolated from patients with chronic venous ulcers (Group no. 1).

**Figure 5:**
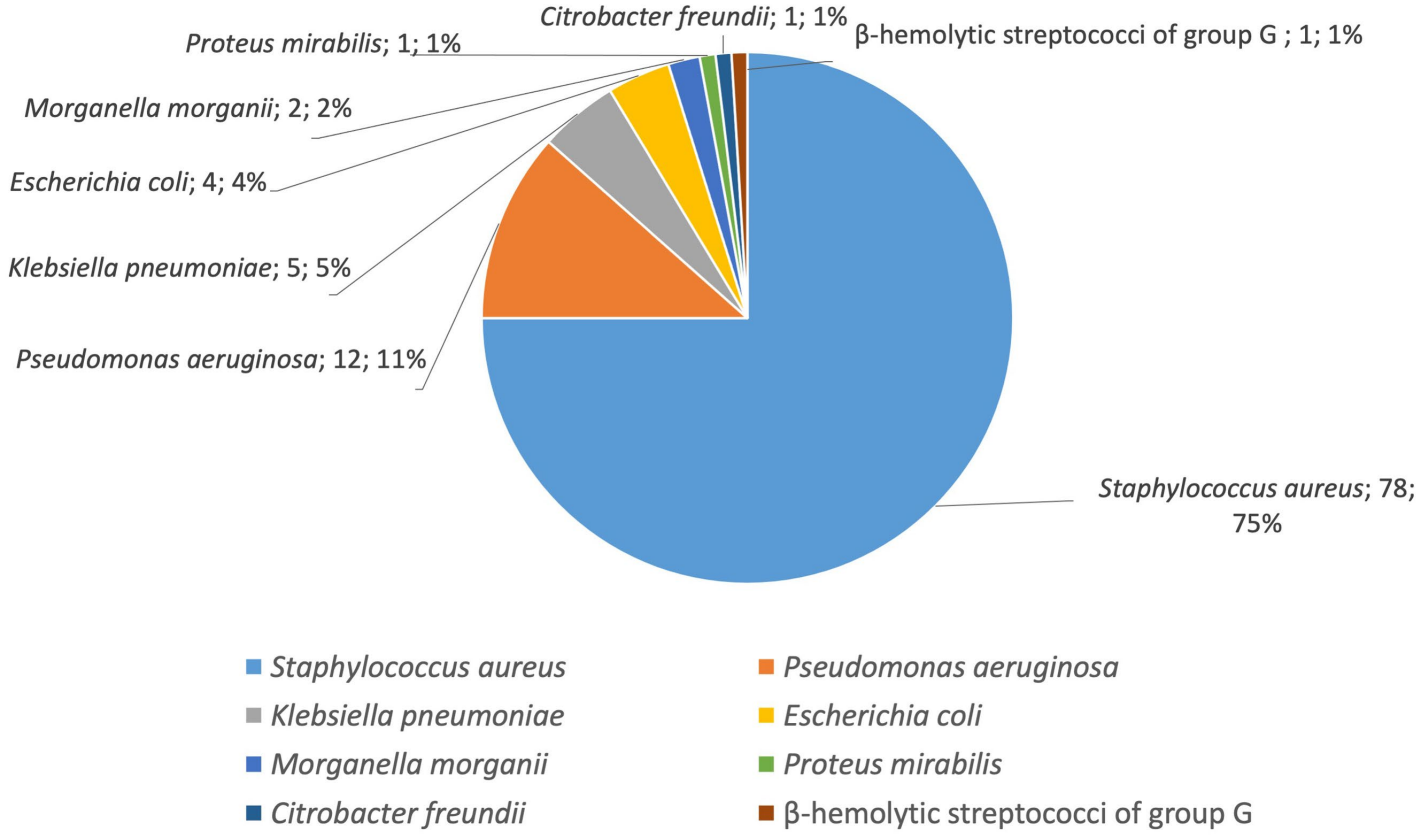
Distribution by species of the isolated bacterial strains (number of strains, %).

During the entire period all 104 isolated strains were identified as belonging to seven bacterial species as follows: the most common was *S. aureus* (78 strains, 75%), followed by *P. aeruginosa* (12 strains, 11%) and the enterobacteria *K. pneumoniae* (5 strains, 5%), *E. coli* (4 strains, 4%), *M. morganii* (2 strains, 2%) *P. mirabilis* (1 strain, 1%), *C. freundii* (1strain, 1%) and β-hemolytic streptococci of group G (1 strain, 1%; Figure 5; Table 4).

**Table 4:** Strains isolated in the study and their sources.

	MSSA	MRSA	*P. aeruginosa*	*K. pneumoniae*	*E. coli*	*M. morganii*	*P. mirabilis*	*C. freundii*	Group G β-hemolytic streptococci
Venous ulcer	21 strains (5, 8, 18, 21, 28, 31, 34, 44, 104, 120, 131, 145, 146, 166, 169, 203, 205, 210, 215, 216, 226)	12 strains (11, 20, 27, 36, 43, 140, 157, 163, 206, 220, 221, 228)	8 strains (7, 16, 37, 39, 108, 164, 207, 213)	2 strains (4, 177)	3 strains (13, 42, 209)	2 strains (214, 218)	0	1 strain (1)	1 strain (9)
Arterial ulcer	5 strains (29, 118, 165, 219, 223)	1 strain (22)	0	0	1 strain (23)	0	1 strain (17)	0	0
Arterio-venous ulcer	0	8 strains (30, 156, 170, 176, 183, 201, 217, 227)	3 strains (143, 181, 202)	0	0	0	0	0	0
Pressure sore	1 strain (35)	4 strains (19, 25, 126, 136)	0	0	0	0	0	0	0
Post-surgical wound	1 strain (14)	1 strain (2)	0	1 strain (3)	0	0	0	0	0
Wounds associated with abscesses	9 strains (6, 10, 26, 32, 33, 38, 40, 41, 117)	5 strains (12, 107, 134, 147, 167)	0	0	0	0	0	0	0
Paraneoplastic ulcerations	2 strains (15, 24)	0	0	0	0	0	0	0	0
Ulcerations in autoimmune vesiculo-bullous diseases	1 strain (204)	7 strains (114, 125, 168, 211, 224, 225, 229)	1 strain (208)	2 strains (180, 212)	0	0	0	0	0

#### 3.2.2 Phenotypic assessment of the virulence profile of microorganisms

##### 3.2.2.1 Phenotypic assessment of the adherence to human cell substrate

Adherence to living or inert substrate and the formation of bacterial aggregates are essential steps in the biofilms’ development (Donlan and Costerton, 2002). Testing the ability of bacterial strains isolated from delayed-healing skin lesions to adhere to human cell substrate was the first step in defining the potential of microorganisms to trigger chronic infections, with an impact on the physiological process of skin healing. For 44 bacterial strains the cell substrate used was HeLa cell line (Figure 1; Table 5), while for the other 60 strains the cellular substrate used was represented by endothelial diploid cell cultures (Table 6).

**Table 5:** Adherence pattern to HeLa cell substrate for each isolated bacterial strain.

Adherence pattern to HeLa cells	No adherence	Localized	Diffuse	Localized aggregative	Diffuse aggregative	Adherence index
*MSSA*	2 strains (35, 38)	4 strains (6, 26, 29, 41)	4 strains (5, 15, 31, 44)	7 strains (8, 10, 18, 24, 28, 33, 40)	4 strains (14, 21, 32, 34)	0–100%
						Average: 52.38%
*MRSA*	0	0	1 strain (11)	5 strains (2, 12, 19, 20, 36)	5 strains (22, 25, 27, 30, 43)	10–100%
						Average: 73.18%
*P. aeruginosa*	0	0	2 strains (7, 39)	0	2 strains (16, 37)	10% (7), 80% (39), 100% (16, 37)
						Average: 63.33%
*C. freundii*	0	0	1 strain (1)	0	0	50% (1)
*E. coli*	1 strain (42)	1 strain (13)	1 strain (23)	0	0	0% (42), 10% (23), 20% (13)
						Average: 10%
*K. pneumoniae*	0	0	0	0	2 strains (3,4)	30% (3), 40% (4)
						Average: 35%
*P. mirabilis*	0	1 strain (17)	0	0	0	5% (17)

MSSA, methicillin-sensitive *Staphylococcus aureus*; MRSA, methicillin-resistant *Staphylococcus aureus*; *P. aeruginosa*, *Pseudomonas aeruginosa*; *E. coli*, *Escherichia coli*; *K. pneumoniae*, *Klebsiella pneumoniae*; *M. morganii*, *Morganella morganii*.

**Table 6:** Adherence pattern to endothelial cell substrate for each isolated bacterial strain.

*Adherence pattern to endothelial cells*	No adherence	Localized	Diffuse	Localized aggregative	Diffuse aggregative	Adherence index
*MSSA*	5 strains (131, 145, 146, 204, 226)	3 strains (118, 166, 169)	3 strains (117, 120, 205)	3 strains (104, 219, 223)	5 strains (165, 202, 203, 210, 215, 216)	0–85%
						Average: 22.42%
*MRSA*	2 strains (125, 225)	6 strains (107, 170, 183, 211, 221, 229)	8 strains (134, 136, 167, 176, 206, 220, 227, 228)	5 strains (114, 126, 147, 201, 217)	6 strains (140, 156, 157, 163, 168, 224)	0–90%
						Average: 31.14%
*P. aeruginosa*	1 strain (207)	0	1 strain (208)	3 strains (108, 164, 213)	3 strains (143, 181, 202)	0–40%
						Average:12.5%
*E. coli*	0	1 strain (209)	0	0	0	5% (209)
*K. pneumoniae*	0	1 strain (189)	0	2 strains (177, 212)	0	Average: 12.66%
						3% (177), 5% (180), 30% (212)
*M. morganii*	0	1 strain (218)	1 strain (214)	0	0	Average: (7.5%)
						5% (218), 10% (214)

MSSA, methicillin-sensitive *Staphylococcus aureus*; MRSA, methicillin-resistant *Staphylococcus aureus*; *P. aeruginosa*, *Pseudomonas aeruginosa*; *E.coli*, *Escherichia coli*; *K. pneumoniae*, *Klebsiella pneumoniae*; *M. morganii*, *Morganella morganii*.

*S. aureus* strains presented an increased adherence capacity to human cell substrate human cell substrate, both for HeLa and endothelial cells, with an average of the adherence index of over 50% for methicillin-sensitive *S. aureus* (MSSA) and over 70% for MRSA, respectively, over 22.42% for MSSA and over 31.14% for MRSA (Tables 2, 3). So, MRSA strains showed the highest adherence to cellular substrate compared to MSSA strains, but also other bacterial strains belonging to other species showed this property.

It was observed that MRSA and *P. aeruginosa* strains showed the highest degree of adherence to the human cell substrate and their possible association in polymicrobial infections could influence the persistence of the infection at the level of venous ulcers.

Compared to *S. aureus* and *P. aeruginosa* strains, the adherence of enterobacteria to human cell substrate was significantly lower quantitatively, the adherence index to HeLa cells being between 0 and 50%, respectively between 0 and 30% to endothelial cells. Their adherence pattern was also suggestive of the strains’ reduced ability to develop bacterial aggregates. If *E. coli* and *P. mirabilis*, isolated from lower limb venous and arterial ulcers adhered locally or diffusely, in minimal proportions to the substrate 0%–20%, the strains of *C. freundii* and *K. pneumoniae*, isolated from venous ulcers, showed a diffuse or diffuse aggregative adherence to HeLa cells of 30–50% (Table 2). This result does not exclude the involvement of *C. freundii* and *K. pneumoniae* in the development of chronic mono- or polymicrobial infections in venous ulcers.

An interesting observation concerns the fact that the strains tested showed a preferential type of adherence depending on the isolation source. The diffuse aggregative subtype of adherence to human cells was observed more frequently in strains isolated from chronic lower limb ulcers (venous or of other etiology), compared to other types of chronic skin lesions (Supplementary Figures 1, 2). This adherence pattern suggests the great potential of microorganisms to develop cellular aggregates and implicitly biofilms.

The adherence index was high for all sources, with the highest values recorded to HeLa cells of 92.5% for lower limb ulcers that exclude venous etiology (Supplementary Figure 3), and to endothelial cells of 60.7%, for venous ulcers (Supplementary Figure 4).

The strains isolated from venous ulcers showed a high degree of adherence to HeLa cell substrate, regardless of the observed adherence pattern. These results are also reflected by the value of the average HeLa index established only according to the isolation source, which was almost two times higher in strains isolated from venous ulcers (46%) compared to those isolated from leg ulcers of other etiology (25%) or other types of chronic skin lesions (25%). The average index of adherence to endothelial cells was also higher in strains isolated from venous ulcers (24%) compared to those isolated from leg ulcers of other etiology (16%) or other types of chronic skin lesions (21%).

##### 3.2.2.2 Phenotypic assessment of biofilm development *in vitro*

All strains of *S. aureus* developed biofilms on the inert substratum, with variations observed depending on the source of isolation. Strains isolated from arterial and artero-venous ulcers showed the lowest ability to form such protective structures. The strains isolated from venous ulcers developed biofilms with a medium intensity, while staphylococci from other types of chronic skin lesions produced biofilms with the highest intensity. The spectrophotometric quantification of the biomass of *S. aureus* did not demonstrate significant differences between MSSA and MRSA isolates from lower limb ulcers, regardless of etiology. On the other side, MRSA strains isolated from other types of chronic skin lesions showed a more intense ability to form biofilms, compared to MSSA strains. Quantitative detection revealed maximum values after culture plate incubation periods of 24 and 48 h. After a 72 h of incubation, the large mass of biofilms becomes more fragile, thus detaching more easily from the surface of the micro-wells during the fixation and staining processes.

All strains of *P. aeruginosa* developed biofilms, with a significant difference between the strains isolated from venous ulcers compared to the strains that came from other sources (they produced more intensively such structures). Strain no. 7 produced the most intense biofilm. Compared to *S. aureus* strains the production of biofilms was similar. Strain no. 16, characterized by an increased ability to develop biofilms, was isolated from a patient diagnosed with chronic venous ulcer, who died of sepsis; this strain showed a complex virulence profile by the production of pore-forming toxins, DN-ase, and by an intense diffuse aggregative adherence to HeLa cells and an adherence index of 100%, as well as multiple antibiotic resistance.

The ability to develop biofilms of *Enterobacteriaceae* strains was significantly lower compared to *S. aureus* and *P. aeruginosa* strains. This result is consistent with the analysis of adherence to HeLa cell or endothelial cell substrate, inferior in *Enterobacteriaceae* compared to *S. aureus* and *P. aeruginosa* strains. Compared to other enterobacteria, *K. pneumoniae* strains showed both greater adhesion to cellular substrate, as well as more intense biofilm development.

Besides testing the ability of each strain to develop biofilms, this virulence characteristic was also evaluated for the polymicrobial association of strains isolated from the same sample. Thus, the development of biofilms was studied for the following microorganisms: 4 bimicrobial associations, 3 of which were represented by *P. aeruginosa* and MRSA and a bimicrobial association of *P. aeruginosa* and MSSA, as well as an association of 3 bacterial species: *P. aeruginosa*, MSSA, and *M. morganii*.

Where MRSA and *P. aeruginosa* strains isolated from arterio-venous lower limb ulcers were inoculated together, the formation of biofilms was observed with a much-increased intensity, up to 5–6 times higher. In polymicrobial infections, *P. aeruginosa* has the ability to increase the virulence of biofilms by enhancing the development of other bacterial species (Scales and Huffnagle, 2013). Regarding the associations of strains originating from venous ulcers, in both bimicrobial and trimicrobial biofilms, no significant increases were observed compared to individual strains.

##### 3.2.2.3 Phenotypic assessment of the production of soluble virulence factors

The phenotypic virulence profiles of different bacterial species were analyzed and compared, by cultivating isolated strains on special media containing the enzyme substrate corresponding to each soluble virulence factor: pore-forming toxins (lecithinase, lipase, hemolysins) and exotoxins (caseinase, gelatinase, amylase, DN-ase), esculinase (Figures 2, 3).

Regarding the distribution of virulence factors in *S. aureus* strains, it was observed that lecithinase was the most frequently expressed virulence factor (62 strains, 80.5%), followed, in approximately equal proportions, by lipase (55 strains, 71.4%), hemolysins (56 strains, 72.7%), and esculinase (56 strains, 72.7%). To a lesser extent, DN-ase (34 strains, 44.2%), caseinase (35 strains, 45.5%), and amylase (32 strains, 41.6%). Gelatinase was the least expressed virulence factor in the tested strains. Thus, *S. aureus* strains predominantly expressed pore-forming toxins (lecithinase, lipase, and hemolysins), necessary for bacterial multiplication and dissemination. Hemolysins are also involved in increasing the supply of iron, necessary for the activation of microbial genes and the expression of other virulence factors (Table 7).

**Table 7:** The distribution of virulence factors in *Staphylococcus aureus* strains.

Soluble virulence factor	*Staphylococcus aureus* (all strains)		MSSA		MRSA	
	No.	%	No.	%	No.	%
Alpha-hemolysin	22	28.6	9	22.5	13	35.1
Beta-hemolysin	34	44.2	22	55.0	12	32.4
Lipase	55	71.4	29	72.5	26	70.3
Lecithinase	62	80.5	35	87.5	27	73.0
Esculinase	56	72.7	26	65.0	30	81.1
DN-ase	34	44.2	24	60.0	10	27.0
Caseinase	35	45.5	16	40.0	19	51.4
Gelatinase	17	22.1	5	12.5	12	32.4
Amylase	32	41.6	21	52.5	11	29.7
Total no. of virulence factors	347		187		160	

MSSA, methicillin-sensitive *Staphylococcus aureus*; MRSA, methicillin-resistant *Staphylococcus aureus*.

Comparing the phenotypic virulence profile of MSSA vs. MRSA strains, significant differences in the production of virulence factors were observed. MSSA strains showed more often complete hemolysis (1.7/1), DNase (2.2/1), amylase (1.7/1), lecithinase (1.2/1) and less often incomplete hemolysis (1/1.5), gelatinase (1/2.6), esculinase (1/1.2), caseinase (1/1.3). Lipase was expressed in approximately equal proportions. MRSA strains, compared to MSSA strains, expressed, along with pore-forming toxins, more intensively the enzymes involved in the degradation of the extracellular matrix, devitalized tissue and debris, such as caseinase and gelatinase (which belongs to matrix metaloproteinases).

The virulence profile for each bacterial species was compared, depending on the source of isolation: venous ulcer, lower limb ulcer of other etiology and other types of chronic skin lesions.

From the point of view of the production of hemolysins, the strains that came from lower limb ulcers of vascular etiology (venous, arterial, mixed), regardless of the bacterial species, showed a more frequent expression of β-hemolysins. Moreover, *Enterobacteriaceae* strains from venous ulcers expressed alpha-hemolysins significantly more frequently compared to the isolated from other sources (75% vs. 50 and 33.3%).

The strains that came from lower limb ulcers of vascular etiology (venous, arterial, mixed), compared to those from other chronic skin lesions, also showed the following differences in terms of the frequency of production of virulence factors: increased production of gelatinase in *Enterobacteriaceae* but lower in *P. aeruginosa*, decreased esculinase production in *Enterobacteriaceae* but increased in *P. aeruginosa*, decreased lecithinase production in *P. aeruginosa*. For strains isolated from venous ulcers, compared to microorganisms that came from other sources, a more frequent expression of lipase and amylase was observed in MRSA and *P. aeruginosa*. For strains of MSSA and *Enterobacteriaceae* this proportion was reversed. DNase was produced only by *P. aeruginosa* strains originating from venous ulcers.

*Pseudomonas aeruginosa* strains isolated from other types of chronic skin lesions intensively produced gelatinase and caseinase, enzymes involved in local invasiveness, since they degrade the extracellular matrix of the connective tissue. The spectrum of soluble virulence factors of *P. aeruginosa* strains isolated from venous ulcers was different from that of strains isolated from other sources, suggesting the existence of some damaging factors that could modify the virulence profile of microorganisms. Moreover, *Enterobacteriaceae* from venous ulcers expressed virulence factors involved in local invasiveness (caseinase, gelatinase), but also in the dissemination of infection (alpha-hemolysins).

According to the average virulence index calculated, a similar intensity of expression of virulence factors was observed in *S. aureus* strains isolated from non-venous lower limb ulcers and from other types of chronic skin lesions. The virulence of MRSA strains isolated from venous ulcers was slightly higher compared to those from other sources (ratio of 1.12/1/1). *Pseudomonas aeruginosa* strains and those belonging to the *Enterobacteriaceae* family isolated from other types of chronic skin lesions had a more intense virulence profile compared to those isolated from venous or leg ulcers.

#### 3.2.3 Genotypic assessment of virulence genes of MRSA strains by polymerase chain reaction

The study characterized the phenotypic and genotypic virulence profile of 24 MRSA selected strains isolated from chronic skin lesions (12 strains from chronic venous ulcers) of patients admitted to the Dermatology Clinic, “Elias” University Emergency Hospital, Bucharest (Table 8).

**Table 8:** Results of the genotypic assessment of 8 virulence genes in MRSA strains isolated from chronic wounds, through Polymerase Chain Reaction uniplex/simplex tests (*fnbA* gene coding, fibronectin adhesin A; *coag* gene, coagulase enzyme) and multiplex tests (*clf*A and *clf*B genes, bacterial surface adhesins; *fnb*B gene, fibronectin binding protein B; *fib* gene, fibrinogen binding protein; *bbp* gene, bone sialoprotein–binding protein; *ebpS*, elastin binding protein; Cotar et al., 2010).

No.	Strain code	Isolation source	Virulence encoding genes							
			*clf A*	*clfB*	*fnbB*	*fib*	*fnbA*	*coag*	*bbp*	*ebpS*
1	11	Venous ulcer	+	+	−	−	−	+	−	−
2	20	Venous ulcer	+	+	−	−	+	+	−	−
3	27	Venous ulcer	+	+	−	−	+	+	−	−
4	36	Venous ulcer	+	+	−	−	+	+	−	−
5	43	Venous ulcer	+	+	−	−	+	+	−	−
6	140	Venous ulcer	+	+	−	−	−	+	−	−
7	157	Venous ulcer	+	+	−	−	−	−	−	−
8	163	Venous ulcer	−	−	−	−	+	+	−	−
9	206	Venous ulcer	+	+	−	−	−	+	−	−
10	220	Venous ulcer	+	+	−	−	+	+	−	−
11	221	Venous ulcer	+	+	−	−	−	+	−	−
12	228	Venous ulcer	+	+	−	−	−	+	−	−
13	114	Wound-autoimmune vesiculo-bullous diseases	+	+	−	−	+	+	−	−
14	136	Pressure sore	+	+	−	−	+	+	−	−
15	156	Arterio-venous ulcer	+	+	−	−	+	+	−	−
16	168	Wound-autoimmune vesiculo-bullous diseases	+	+	−	−	+	+	−	−
17	176	Arterio-venous ulcer	+	+	−	−	+	+	−	−
18	183	Arterio-venous ulcer	+	+	−	−	+	+	−	−
19	2	Surgical wound	+	+	−	−	+	+	−	−
20	12	Wound associated with abscess	+	+	−	−	+	+	−	−
21	19	Pressure sore	+	+	−	−	+	+	−	−
22	22	Arterial ulcer	+	+	−	−	+	+	−	−
23	25	Pressure sore	+	+	−	−	−	+	−	−
24	30	Arterio-venous ulcer	+	+	−	−	+	+	−	−

The studied strains showed a variable ability to adhere to the cell substrate and develop biofilms, observing a statistically significant linear correlation between the two studied properties. The analysis of the distribution of extracellular virulence factors demonstrated that the pore-forming toxins, hemolysins, lipase, and lecithinase, were produced, each in 75% of the MRSA strains analyzed, in approximately equal proportions depending on the location source. This highlights the increased potential for systemic dissemination of MRSA strains. Caseinase and gelatinase exoproteases were produced by a smaller number of MRSA strains (45.8% and 25%, respectively). DNase was detected in 32% of the strains.

Phenotypic changes are most often correlated with a specific genotypic profile. So, the next experimental step was represented by genotypic screening aimed at identifying the presence or absence of certain genes encoding extracellular virulence factors or involved in invasiveness and adhesion.

The analysis of the distribution of virulence genes demonstrated that 23 of the 24 analyzed strains (95.8%) expressed genes encoding adhesins associated with the bacterial surface (*clfA* and *clfB*), 23 strains expressed the *coag* gene (95.8%), and 17 strains had expressed *fnbA* gene (70.8%). The *fnbA* gene was less frequently detected in strains isolated from venous ulcers, in a proportion of 50%, compared to 91.6% in the case of other sources of isolation (91.6%; arterial ulcers, arterio-venous ulcers, chronic surgical wounds, chronic abscesses). None of the MRSA strains analyzed expressed the genes *fnbB* (fibronectin-binding protein), *fib, bbp* and *ebpS* (elastin-binding protein; Table 8).

### 3.3 Statistical analysis

No statistically significant correlation was found between the cell substrate adherence index of the strains isolated from venous ulcers and the initial RESVECH severity score of the wounds (Pearson’s r = −0.033). Also, no positive or negative correlation was detected between the detection of a polymicrobial infection during the microbiological diagnostic examination and the ability of the isolated microorganisms to adhere to the cell substrate (Pearson’s r = 0.064).

The test of the linear correlation between the index of adherence to the cell substrate and the average intensity of development of bacterial biofilms of the strains isolated from venous ulcers revealed a Pearson r coefficient of 0.456 (>0.2) and a statistically significant correlation. Thus, it was demonstrated that the adherence of bacterial strains to human cellular substrate correlates proportionally with their property to develop biofilms.

The following values were associated with each adherence pattern: localized adherence = 1, diffuse adherence = 2, aggregative localized adherence = 3, aggregative diffuse adherence = 4, depending on their potential to be associated with the development of biofilms. The test of the linear correlation between the pattern of adherence to the cell substrate and the average intensity of development of bacterial biofilms of the strains isolated from venous ulcers revealed a statistically significant correlation, with a Pearson r coefficient of 0.339. It was thus demonstrated that the pattern of adherence to the cellular substrate, including the property of microorganisms to form cellular aggregates, correlated proportionally with the property of MRSA to develop biofilms.

Statistical tests revealed a weak positive correlation between the average intensity of bacterial biofilm development of strains isolated from venous ulcers and the initial RESVECH severity score of the lesions, with a Pearson r coefficient of 0.203 < 0.28. Considering the multitude of factors that influence the severity of venous ulcers such as host comorbidities, vascular deficiency, the correctness of treatment administration, the type of treatment used and many others, this result does not exclude the relevance of the pathogenic hypothesis. Further studies are needed to establish whether there is a correlation between the intensity of bacterial biofilm development and the severity of the venous ulcer lesion.

There was no correlation between the mean intensity of development of bacterial biofilms in strains isolated from venous ulcers and the final evolution of the patients (healing = 1, improvement = 2, stagnation = 3, aggravation = 4, death = 5; Pearson’s r = −0.011). The evolution of patients’ injuries can be influenced by multiple factors, including the correctness of administering the recommended treatment at home.

## 4 Discussion

In an unique attempt in international scientific research, within the experimental study, we characterized phenotypically and molecularly the virulence profiles of bacterial strains isolated from chronic venous ulcers, as well as from other types of chronic wounds associated with difficult to treat infections. We aimed to discover clinical-microbiological correlations, obtaining results that might contribute to the elucidation of the impact of the microbiota on delayed wound healing.

Studies of the cutaneous microbiome conducted *in vitro* and *in vivo* have confirmed the widespread belief that the microorganisms colonizing skin wounds affect their healing. Contradictory results have emerged from these studies, nevertheless. In their *in vivo* study on germ-free Swiss mice, Canesso et al. (2014) showed rapid and scarless wound healing in the absence of commensal skin microbiota. Additionally, neutrophil accumulation was decreased, anti-inflammatory cytokine levels were elevated, and vascular endothelial growth factor (VEGF) was upregulated (Canesso et al., 2014). Their wound healing characteristics resembled those of standard mice after the skin microbiota had been restored, indicating that the commensal bacteria might have a negative impact (Canesso et al., 2014), because they, especially staphylococci, can behave as opportunistic pathogens. On the other hand, it was discovered that animals receiving oral Vancomycin had a lower bacterial density of skin wounds and slower wound healing rates due to the impact on keratinocyte differentiation and proliferation by the downregulation of the proteins interleukin (IL)-17 and regenerating islet derived protein-III gamma (RegIIIy; Zhang et al., 2015; Johnson et al., 2018).

Bacteria are able of intercellular communication and further, to sense and rapidly adapt to their environment through coordinated, multi-cellular responses. Even in human wounds, they are able to sense, respond, and manipulate host-immune responses, toward local persistence of infection and, in particular circumstances, toward systemic invasion and septicemic events (Chifiriuc et al., 2022).

The evolution of patients with chronic, superinfected venous ulcers was less severe compared to that of patients with decubitus ulcers or chronic leg ulcers of other etiology (arterial or mixed). This result could be determined by the existence of multiple decompensated comorbidities in the latter, but also by different bacterial virulence phenotypes.

Adherence to living or inert substrate and the production of bacterial aggregates are essential steps in the development of biofilms (Donlan and Costerton, 2002). Testing the ability of bacterial strains isolated from delayed-healing wounds to adhere to human cellular substrate was the first step in defining the potential of microorganisms to trigger chronic infections, with an impact on the physiology of skin healing.

Bacterial strains isolated from chronic venous ulcers, but also from other types of chronic lower limb ulcers, showed high intensity adherence to human cellular substrate, of diffuse aggregative type, statistically associated with the microorganisms’ ability to develop bacterial biofilms (Pearson r 0.456 > 0.28).

MRSA strains showed a more significant ability to adhere and aggregate to human cell substrate (both HeLa cells and endothelial cells) compared to other bacterial species studied and, implicitly, an increased potential to develop bacterial biofilms. Along with the multi-resistance of MRSA to antibiotics, the increased tolerance of biofilms to the action of antimicrobial molecules may further reduce the variety of effective therapeutic options. It was observed that MRSA and *P. aeruginosa* strains showed the highest degree of adherence to the cellular substrate and their possible association in polymicrobial infections could influence the persistence of infection in venous ulcers, as well as other types of wounds.

Compared to *S. aureus* and *P. aeruginosa* strains, the adherence of the studied enterobacteria to the HeLa or endothelial cell substrates was significantly less quantitatively. A possible explanation may be that the planktonic, free phenotype is probably more characteristic for these microorganisms. The presence of biological inflammatory syndrome in patients with *Enterobacteriaceae* wound isolates could be explained precisely by the potential of the planktonic phenotype to trigger a more intense inflammatory response. *Klebsiella pneumoniae* strains, being capsulated, were the most adherent enterobacteria.

An interesting observation is the identification of a particular pattern of adherence depending on the isolation source. The strains isolated from chronic lower limb ulcers of vascular etiology, compared to chronic skin lesions of other etiology, were more frequently associated with the diffuse aggregative pattern of adherence, of high intensity, to both types of cells used as substrate. This adherence pattern suggests the maximum potential of microorganisms to develop cellular aggregates and implicitly biofilms. Moreover, the strains isolated from venous ulcers showed a high degree of adherence to the cellular substrate for each pattern observed, more intense compared to the strains isolated from leg ulcers of other etiology or other types of chronic skin lesions. These results may explain the persistence of the infection and the reduced response to antimicrobials.

Adapting therapeutic management by using molecules that disrupt the adherence of microorganisms to the cellular substrate within lower limb ulcers could contribute to a lower infectious burden and faster healing rates.

Bacterial adherence to the endothelial cell substrate *in vitro* has been shown to be involved both in the systemic dissemination of infections and in the etiopathogenic mechanisms of severe conditions such as acute endocarditis (Ogawa et al., 1985; Claes et al., 2018). Our study revealed that the adherence to the endothelial cell substrate of the strains isolated from chronic skin lesions, regardless of etiology, was much reduced compared to the adhesion to the HeLa cell substrate. This could explain the rarity of developing severe systemic infections in patients with chronic wounds. However, MRSA strains showed a more significant ability to adhere and aggregate to the endothelial cell substrate compared to other bacterial species studied and therefore we emphasize the increased risk of the patients included in the study to develop severe infectious complications, being elderly patients, with multiple cardiovascular comorbidities.

The next stage of the study was represented by testing the ability of microorganisms to produce biofilms, structures compared by some authors to some “multi-cellular, primitive organisms” (Hoiby et al., 2010). Bacterial biofilms are “structurally and dynamically complex biological systems” (Lurie et al., 2020), composed of mono- or polymicrobial communities embedded in a protective extracellular matrix, which gives bacteria tolerance to antibiotics and to host’s immune defense mechanisms (Mihai et al., 2015). The main characteristic of chronic wounds is the unfavorable evolution, even if the treatment regimen is in accordance with international guidelines. Most studies support a correlation between the presence of bacterial biofilms and the chronicity of skin lesions, but there are authors who doubt this hypothesis (Thomson, 2011; Percival et al., 2012, 2014).

All isolated strains demonstrated the ability to develop biofilms, with a variable intensity depending on the bacterial species, but also on the source of isolation. For the strains isolated from venous ulcers, there was not identified a significant correlation between the average intensity of development of bacterial biofilms and the final evolution of the wounds, that may be influenced by multiple factors, including the compliance to treatment recommended at home.

All strains of *S. aureus* developed biofilms, with variations observed depending on the source of isolation. *Staphylococcus aureus* strains isolated from venous ulcers developed biofilms with a higher intensity compared to the strains from lower limb ulcers of arterial or mixed etiology. No significant differences were observed between MRSA and methicillin-susceptible strains.

*Pseudomonas aeruginosa* strains developed biofilms intensively but those isolated from venous ulcers produced these structures more intensively compared to the strains that came from other sources. The ability to develop biofilms of *Enterobacteriaceae* strains was significantly lower compared to *S. aureus* and *P. aeruginosa* strains.

Pluri-microbial inoculations of MRSA and *P. aeruginosa* strains with the same source of infection (leg ulcers of mixed etiology, chronically superinfected inverse psoriasis) were associated with a 5–6 times higher intensity of biofilm development compared to individual strains.

*Pseudomonas aeruginosa* showed less virulent phenotypes. Both the ability to develop biofilms of strains from the *Enterobacteriaceae* family and the degree of virulence were inferior to microorganisms from the species *S. aureus* and *P. aeruginosa*.

In the next step we analyzed and compared the phenotypic virulence profiles in different bacterial species, by cultivating the isolated strains on special media containing the enzyme substrate corresponding to each soluble virulence factor: pore-forming toxins (lecithinase, lipase, hemolysins) and exoenzymes (caseinase, gelatinase, amylase, DN-ase, esculinase). While the characteristics of invasiveness, tissue destruction, and infection dissemination are primarily associated with the soluble virulence factors secreted by bacteria, the development of biofilms is primarily associated with the persistence of infection, resistance, and tolerance to antimicrobials, as well as host immune defense mechanisms (Preda et al., 2021). The manifestation of these virulence traits may account for the severity of wound infections as well as for their chronicization, which makes them challenging to cure (Preda et al., 2021).

Pore-forming toxins such as lecithinase, lipase, hemolysins are involved in the mechanisms of spreading the infection. Lecithinases or phospholipases give microorganisms the ability to destroy human tissues by degrading lecithin (phosphatidylcholine) into phosphorylcholine and an insoluble diglyceride (Lazar and Balotescu, 2004; Holban et al., 2013).

By hydrolyzing starch, amylase causes the release of glucose, a carbon source useful for microorganisms in intracellular metabolic processes and, implicitly, in triggering bacterial pathogenic mechanisms; this is not significant for skin bacteria (Lazar and Balotescu, 2004; Chifiriuc and Lazar, 2011; Holban et al., 2013). It has been demonstrated that microorganisms lose their ability to multiply and express virulence factors in the absence of iron, an essential element in the development of these mechanisms of bacterial pathogenicity (Lazar and Balotescu, 2004; Chifiriuc and Lazar, 2011; Holban et al., 2013). When the extracellular environment is deficient in iron, to ensure the necessary intake, microorganisms express different virulence factors such as esculinase and bacterial hemolysins (Lazar and Balotescu, 2004; Chifiriuc and Lazar, 2011; Alina-Maria Holban et al., 2013). Esculetol has high affinity for iron and bacterial production of esculinase thus ensures the formation of iron stores necessary for the activation of microbial genes and the expression of other virulence factors (Lazar and Balotescu, 2004; Chifiriuc and Lazar, 2011; Holban et al., 2013). Hemolysins, by degrading hemoglobin, increase the concentration of iron in the environment and indirectly contribute to bacterial pathogenicity.

Caseinase, a proteolytic enzyme, intervenes in invasiveness by damaging the extracellular matrix of the connective tissue (Veis et al., 1997; Holban et al., 2013). Gelatinase is also an enzyme with a broad proteolytic spectrum (Holban et al., 2013; Ionescu et al., 2015).

In the conducted study, *S. aureus* strains predominantly expressed pore-forming toxins (lecithinase, lipase and hemolysins), necessary for systemic infectious dissemination. Moreover, MRSA strains compared to MSSA strains expressed, along with pore-forming toxins, also enzymes involved in the degradation of extracellular matrix proteins and cellular debris, such as caseinase and gelatinase.

Analysis of the phenotypic virulence profile for *P. aeruginosa* strains revealed an intense ability to express pore-forming toxins and exoenzymes. Compared to *S. aureus*, *P. aeruginosa* expressed in a higher proportion the proteases gelatinase and caseinase, involved in local invasiveness, but also hemolysins involved in dissemination. The strains belonging to the *Enterobacteriaceae* family produced virulence factors in a lower proportion compared to *S. aureus* and *P. aeruginosa* strains.

The strains isolated from venous ulcers expressed virulence factors involved in local invasiveness (caseinase, gelatinase), but also in the dissemination of infection (hemolysins). The virulence of MRSA strains isolated from venous ulcers was slightly higher compared to those from other sources. The strains of *P. aeruginosa* and those belonging to the *Enterobacteriaceae* family isolated from lower limb ulcers had a lower virulence profile than chronic skin lesions of other etiology. The spectrum of soluble virulence factors of *P. aeruginosa* strains isolated from venous ulcers was different from that of strains isolated from other sources, suggesting the existence of some damaging factors that could modify the virulence profile of the microorganisms.

Patients with chronic venous ulcers had more frequent recurrences compared to patients suffering from leg ulcers of different etiology. A possible explanation is represented by the persistence of infections with bacterial strains that intensively develop biofilms, through the increased tolerance of these structures to antimicrobial substances or to the host’s defense mechanisms.

MRSA strains possess various pathogenic mechanisms involved in infectious persistence, proving a high adaptability to the conditions in the external environment. Analysis of the phenotypic and genotypic virulence profile of MRSA strains isolated from delayed-healing wounds revealed several interesting results.

In comparison to other studied bacterial species, MRSA strains showed a more intense ability to adhere and aggregate to human cell substrate with the development of biofilms. The increased tolerance of biofilms to the action of antimicrobial molecules may further reduce the variety of effective therapeutic options in MRSA infections. This superior adherence and aggregation capacity may be explained by the detection in the vast majority of the studied strains of *clfA* and *clfB* genes, which encode adhesins or “clumping factors.” These factors mediate the binding of *S. aureus* to fibrinogen and the initiation of infections from skin wounds, the adhesion of microorganisms to eukaryotic cells, but also to the surface of medical devices, thus causing nosocomial infections (Elgalai and Foster, 2003). These adhesins are included in the MSCRAMM (*microbial surface components recognizing adhesive matrix molecules*) category, along with protein A, fibronectin binding proteins (*FnbpA*, *FnbpB*), collagen binding protein and bone sialoprotein binding protein (Chifiriuc and Lazar, 2011).

MRSA strains were also more virulent compared to MSSA. Moreover, the virulence of MRSA strains isolated from venous ulcers was slightly higher compared to microorganisms from other sources.

Adhesion to fibronectin, mediated by the products of the *fnbA* and *fnbB* genes, is important in the adherence of bacteria to endothelial cells, thus being involved in the systemic dissemination of infections (Elgalai and Foster, 2003). Interestingly, the expression of the *fnbA* gene was associated with an increase in the degree of invasiveness of microorganisms and the transition from commensal colonization to systemic infection (Jenkins et al., 2015). In the present study, the *fnbA* gene was detected less frequently in MRSA strains isolated from venous ulcers, compared to other sources of isolation (arterial ulcers, mixed ulcers, chronic surgical wounds, chronic abscesses). This result could explain the rarity of severe systemic infectious events in patients with chronic venous disease.

The presence of a gene does not necessarily imply its phenotypic expression. For example, adhesion to fibronectin may vary, even if *fnbA* or *fnbB* are detected by PCR (Elgalai and Foster, 2003). Groups of researchers have observed that *S. aureus* strains show great diversity in their ability to adhere to different proteins (fibrinogen, fibronectin, collagen, laminin; Elgalai and Foster, 2003). Moreover, *S. aureus* expresses virulence factors differently, depending on the isolation source: the strains isolated by blood culture or from the peritoneal fluid being more virulent compared to those isolated from the tear secretion or from the nasal mucosa (Ionescu et al., 2015).

The *coag* gene encodes an extracellular protein, coagulase, which has two functions: fibrin clot formation by converting fibrinogen to fibrin and plasma coagulation by binding to prothrombin (Entenza et al., 2000). Thus, the microorganisms are protected from the action of the host’s bactericidal molecules or from phagocytosis (Chifiriuc and Lazar, 2011). The belonging to the *aureus* species of *Staphylococccus* strains is confirmed by the coagulase production test. In the vast majority of the strains studied, the *coag* gene was detected (95.8%), involved in the protection of microorganisms from the action of the host’s bactericidal molecules or from phagocytosis. It represents a third mechanism by which these microorganisms persist in chronic wounds, along with antibiotic resistance and biofilm tolerance to antibacterial agents.

*EbpS* encodes the elastin-binding protein, which gives microorganisms the property to adhere to elastin-rich tissues such as skin or lung tissue. Ionescu et al. (2015) reported the presence of the *Ebps* gene in 71.5% of a total of 144 MRSA strains isolated from nasal, ocular, skin wound secretions, peritoneal fluid or isolated by blood culture, and *clfA* and *clfB* genes were present in 99% of the strains. This gene was not detected in the strains studied in this research, suggesting the involvement of other mechanisms in the adhesion of MRSA isolated from chronic skin wounds to the cellular substrate.

This study is subject to several limitations that could be addressed in future research.

First of all, one limitation is represented by the heterogeneity in the study group. Patients diagnosed with chronic wounds usually suffer from cardiovascular diseases, obesity, diabetes mellitus, and other pathologies, with various degrees of severity, that may impact the prognosis of wound healing. One solution may be to include in future studies a larger number of patients randomly stratified by comorbidities and other individual factors.

Another limitation of the study is represented by the diagnosis heterogeneity in the control group. In future research we aim to restrict the inclusion criteria toward one particular disease in the control group (e.g., acute wound, surgical wound).

The follow-up may also show particular limitations. Although it was made at a standardized period of time, chronic wounds can be significantly impacted by multiple factors such as: compliance to treatment, hygiene conditions at home, management of comorbidities in an elderly population. Probably a better approach may be to reduce the follow-up to 1 week of hospitalization, where, under continuous medical supervision, a standard treatment can be kept more strictly and there can be obtained a better control of the comorbidities.

One of the main study limitations is represented by the use of traditional aerobic culturing techniques in the diagnosis of wound colonization or infection. Moreover, common bacteria, less virulent (coagulase-negative staphylococci) were excluded from the study. The study of commensal bacteria, as well as of the species isolated in anaerobic conditions may contribute to a superior knowledge on wound pathophysiology and on the complex host-microbiome interactions. In future research, in order to reveal the wounds microbiome diversity, we aim to perform both the aerobic and anaerobic microbiologic diagnosis. Moreover, depending on the funding, in order to reveal the diversity and architecture of mono- or poly-microbial biofilms, molecular tests may also be employed such as high-throughput sequencing, biofilm imaging with live/dead cells stains, fluorescence *in situ* hybridization (FISH) in combination with confocal laser scanning microscopy (CLSM).

Due to funding limitations, there was performed the genotypic characterization of the virulence profile only for MRSA strains, without including methicillin-sensitive staphylococci. In future studies we aim to expand the genotypic virulence assays for *Staphylococcus aureus* strains, as well as for other bacterial species.

## 5 Conclusion

The study revealed important differences regarding the clinical evolution and virulence profiles of microorganisms isolated from lower limb wounds, as well as between patients diagnosed with chronic venous ulcers and those with lesions of different etiology. The early identification of high-risk patients suffering of chronic venous ulcers, based on clinical, biological, and microbiological characteristics, may guide toward a personalized approach and, therefore, an optimized outcome. While most of the studies presented in the scientific literature focus on clinical prognostic markers of disease, there is still scarce information on the association of the virulence profile of microorganisms isolated from chronic venous ulcers with disease activity. Host-microbiome interplay and, implicitly, bacterial fitness, may lead to relevant phenotypic and genotypic traits of bacterial virulence, correlated with wound severity and response to therapy.

## Data availability statement

The original contributions presented in the study are included in the article/Supplementary material, further inquiries can be directed to the corresponding authors.

## Ethics statement

The studies involving humans were approved by Ethics Commission of the “Elias” University Emergency Hospital, Bucharest. The studies were conducted in accordance with the local legislation and institutional requirements. The participants provided their written informed consent to participate in this study.

## Author contributions

MM: Funding acquisition, Investigation, Project administration, Visualization, Writing – original draft, Writing – review & editing. MP: Investigation, Writing – review & editing. AH: Investigation, Visualization, Writing – original draft, Writing – review & editing. IG-B: Investigation, Writing – review & editing. LP: Investigation, Writing – original draft. M-CC: Investigation, Writing – review & editing. CG: Writing – review & editing. CB: Investigation, Writing – review & editing. CC: Investigation, Writing – review & editing. VL: Funding acquisition, Project administration, Writing – original draft, Writing – review & editing.
