# Supplementary material for: Clinical and microbiological features of host-bacterial interplay in chronic venous ulcers versus other types of chronic skin ulcers
Source: Front Microbiol. 2024 Feb 5;14:1326904. doi: 10.3389/fmicb.2023.1326904 (PMC10875999; doi:10.3389/fmicb.2023.1326904)
Supplement: Supplementary file 1 [file Data_Sheet_1.docx]

Supplementary Material

# Supplementary Data -

# Supplementary Figures and Tables

## Supplementary Figures


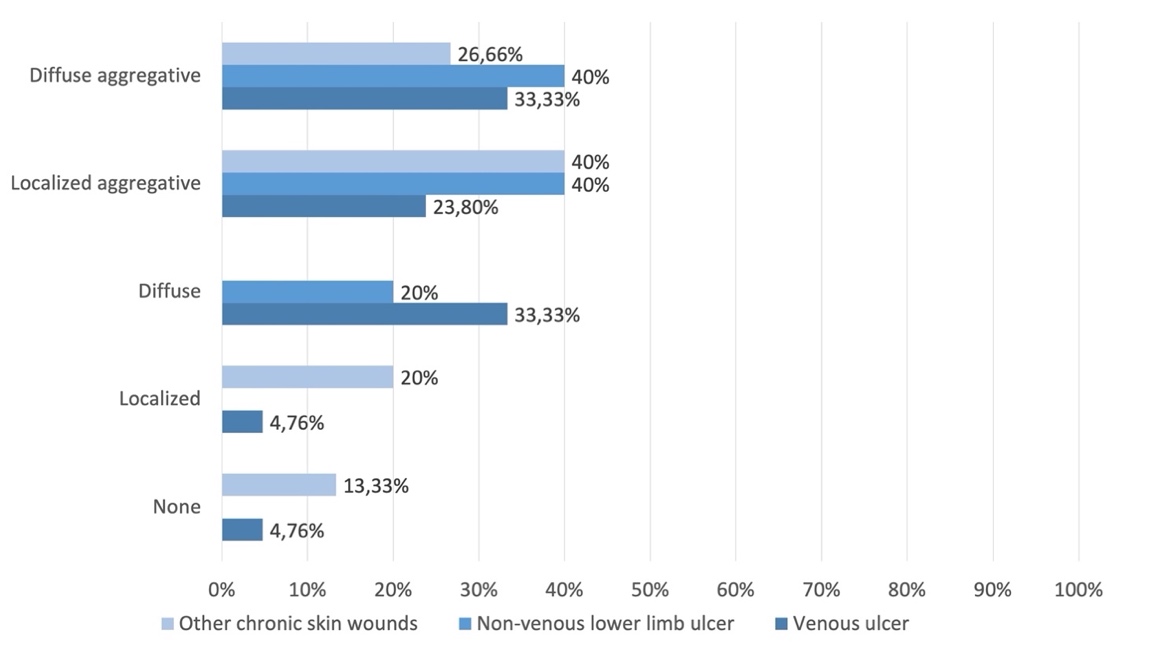


Figure 1. Distribution of the patterns of adherence to the HeLa cell substrate, depending on the source of isolation.


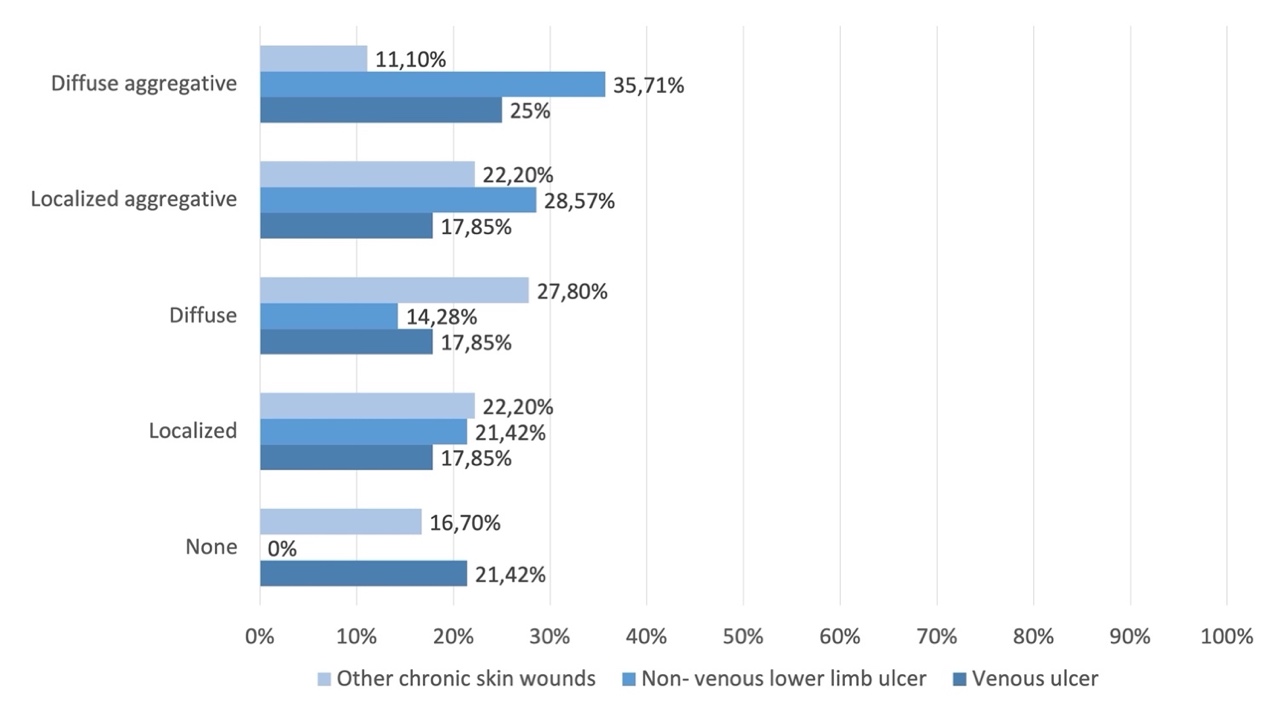


Figure 2. Distribution of the patterns of adherence to the endothelial cell substrate, depending on the source of isolation.


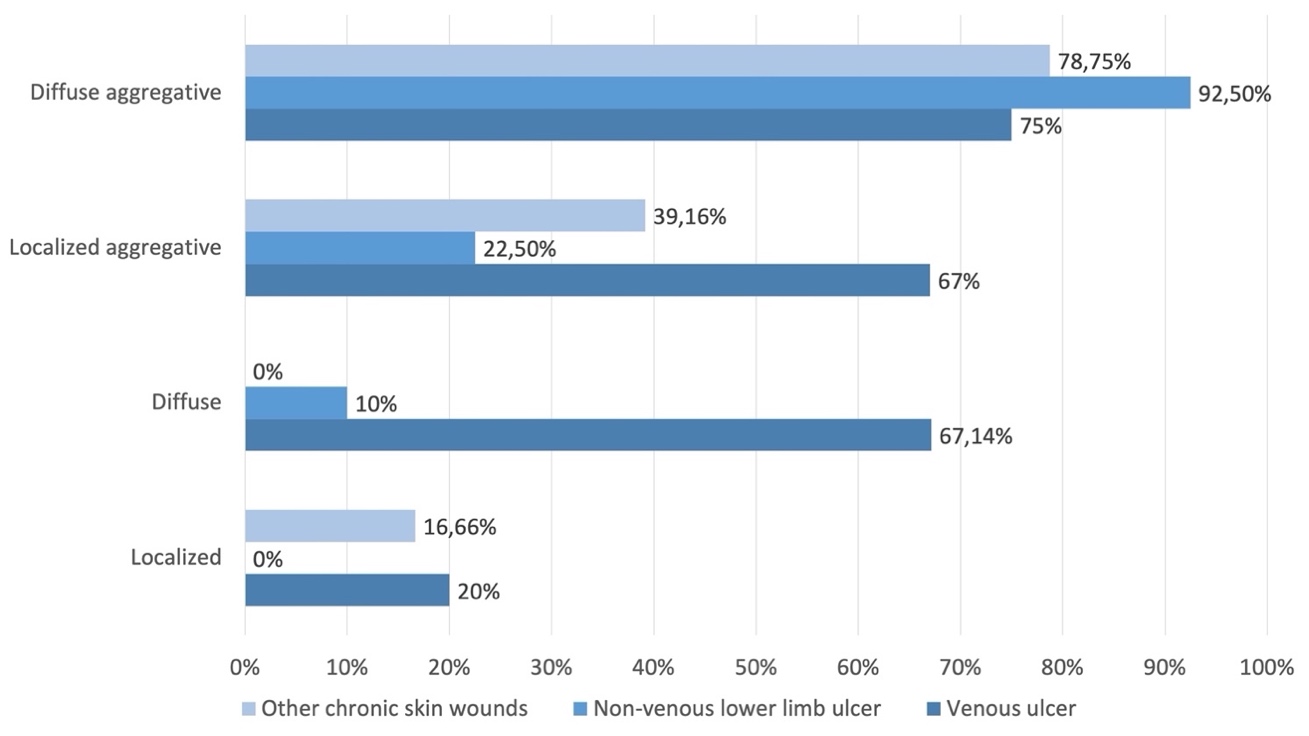


Figure 3. Mean HeLa adherence index depending on the adherence pattern and source of bacterial strain isolation.


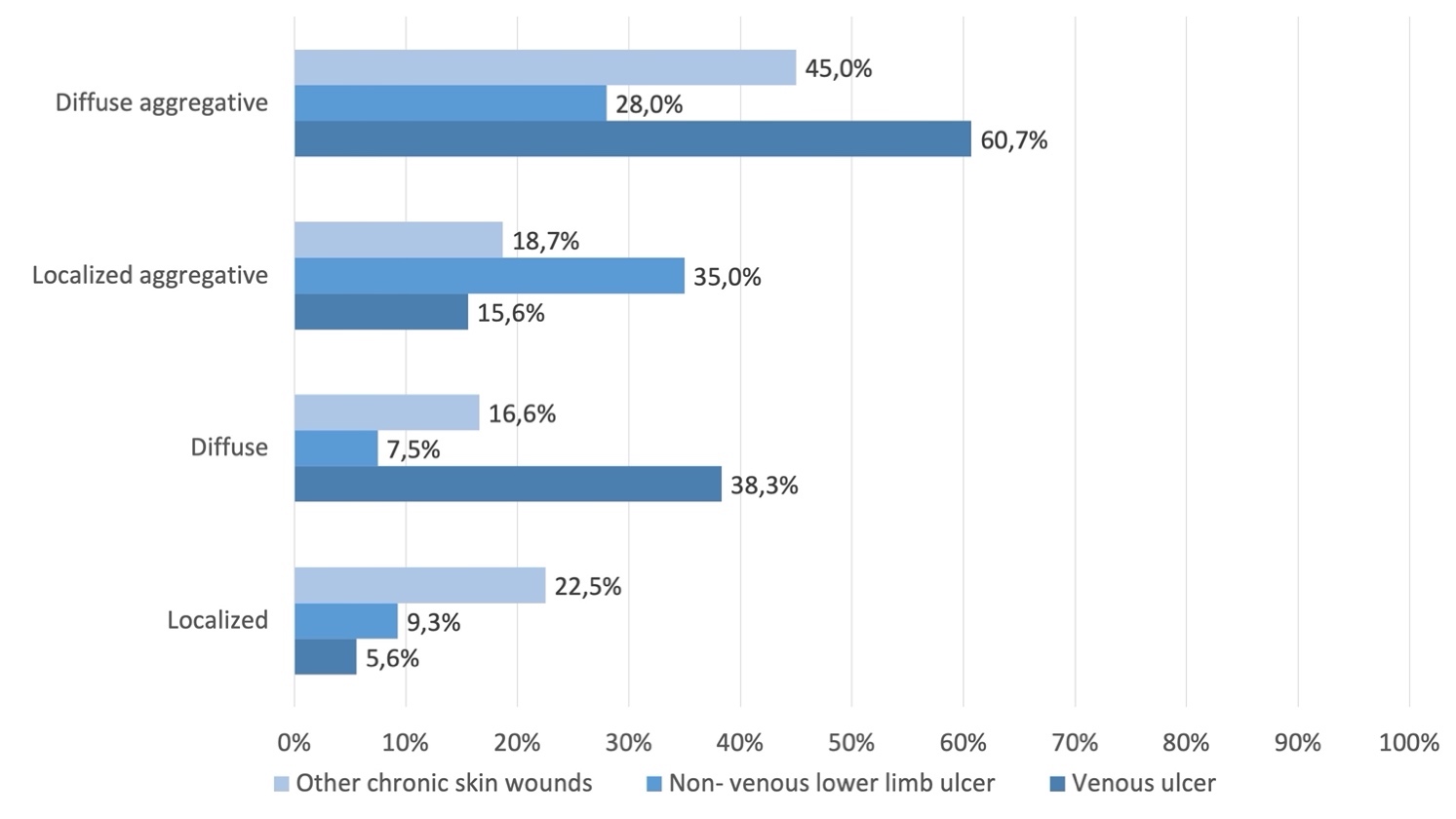


Figure 4. Mean endothelial adherence index depending on the adherence pattern and source of bacterial strain isolation.

2.2. Supplementary Tables

Table 1. Adherence to HeLa cell substrate of the tested strains.

| No. | No. of strain | Species | Wound type | Pattern of adherence | Adherence Index |
| --- | --- | --- | --- | --- | --- |
| 1 | **12** | MRSA | Wound associated with abscess | Localized aggregative | 90 |
| 2 | 32 | *Staphylococcus aureus* | Wound associated with abscess | Diffuse aggregative | 100 |
| 3 | 33 | *Staphylococcus aureus* | Wound associated with abscess | Localized aggregative | 60 |
| 4 | 38 | *Staphylococcus aureus* | Wound associated with abscess | - | 0 |
| 5 | 40 | *Staphylococcus aureus* | Wound associated with abscess | Localized aggregative | 5 |
| 6 | 41 | *Staphylococcus aureus* | Wound associated with abscess | Localized | 25 |
| 7 | 6 | *Staphylococcus aureus* | Wound associated with abscess | Localized | 15 |
| 8 | 10 | *Staphylococcus aureus* | Wound associated with abscess | Localized aggregative | 40 |
| 9 | 26 | *Staphylococcus aureus* | Wound associated with abscess | Localized | 10 |
| 10 | 19 | MRSA | Pressure sore | Localized aggregative | 30 |
| 11 | 25 | MRSA | Pressure sore | Diffuse aggregative | 100 |
| 12 | 35 | *Staphylococcus aureus* | Pressure sore | - | 0 |
| 13 | 2 | MRSA | Surgical wound | Localized aggregative | 10 |
| 14 | 14 | *Staphylococcus aureus* | Surgical wound | Diffuse aggregative | 85 |
| 15 | 22 | MRSA | Arterial ulcer | Diffuse aggregative | 95 |
| 16 | 29 | *Staphylococcus aureus* | Arterial ulcer | Localized | 40 |
| 17 | 30 | MRSA | Arterio-venous ulcer | Diffuse aggregative | 90 |
| 18 | 15 | *Staphylococcus aureus* | Paraneoplastic ulcerations | Diffuse | 100 |
| 19 | 24 | *Staphylococcus aureus* | Paraneoplastic ulcerations | Localized aggregative | 60 |
| 20 | 11 | MRSA | Venous ulcer | Diffuse | 80 |
| 21 | 20 | MRSA | Venous ulcer | Localized aggregative | 70 |
| 22 | 27 | MRSA | Venous ulcer | Diffuse aggregative | 80 |
| 23 | 36 | MRSA | Venous ulcer | Localized aggregative | 70 |
| 24 | 43 | MRSA | Venous ulcer | Diffuse aggregative | 90 |
| 25 | 28 | *Staphylococcus aureus* | Venous ulcer | Localized aggregative | 50 |
| 26 | 31 | *Staphylococcus aureus* | Venous ulcer | Diffuse | 70 |
| 27 | 34 | *Staphylococcus aureus* | Venous ulcer | Diffuse | 90 |
| 28 | 44 | *Staphylococcus aureus* | Venous ulcer | Diffuse aggregative | 15 |
| 29 | 5 | *Staphylococcus aureus* | Venous ulcer | Diffuse | 90 |
| 30 | 8 | *Staphylococcus aureus* | Venous ulcer | Localized aggregative | 60 |
| 31 | 18 | *Staphylococcus aureus* | Venous ulcer | Localized aggregative | 85 |
| 32 | 21 | *Staphylococcus aureus* | Venous ulcer | Diffuse aggregative | 100 |
